# Regulation of *Caenorhabditis elegans* p53/CEP-1–Dependent Germ Cell Apoptosis by Ras/MAPK Signaling

**DOI:** 10.1371/journal.pgen.1002238

**Published:** 2011-08-25

**Authors:** Rachael Rutkowski, Robin Dickinson, Graeme Stewart, Ashley Craig, Marianne Schimpl, Stephen M. Keyse, Anton Gartner

**Affiliations:** 1 Wellcome Trust Centre for Gene Regulation and Expression, College of Life Sciences, University of Dundee, Dundee, United Kingdom; 2 Cancer Research UK Stress Response Laboratory, Medical Research Institute, Ninewells Hospital and Medical School, Dundee, United Kingdom; University of California Davis, United States of America

## Abstract

Maintaining genome stability in the germline is thought to be an evolutionarily ancient role of the p53 family. The sole *Caenorhabditis elegans* p53 family member CEP-1 is required for apoptosis induction in meiotic, late-stage pachytene germ cells in response to DNA damage and meiotic recombination failure. In an unbiased genetic screen for negative regulators of CEP-1, we found that increased activation of the *C. elegans* ERK orthologue MPK-1, resulting from either loss of the *lip-1* phosphatase or activation of *let-60* Ras, results in enhanced *cep-1*–dependent DNA damage induced apoptosis. We further show that MPK-1 is required for DNA damage–induced germ cell apoptosis. We provide evidence that MPK-1 signaling regulates the apoptotic competency of germ cells by restricting CEP-1 protein expression to cells in late pachytene. Restricting CEP-1 expression to cells in late pachytene is thought to ensure that apoptosis doesn't occur in earlier-stage cells where meiotic recombination occurs. MPK-1 signaling regulates CEP-1 expression in part by regulating the levels of GLD-1, a translational repressor of CEP-1, but also via a GLD-1–independent mechanism. In addition, we show that MPK-1 is phosphorylated and activated upon ionising radiation (IR) in late pachytene germ cells and that MPK-1–dependent CEP-1 activation may be in part direct, as these two proteins interact in a yeast two-hybrid assay. In summary, we report our novel finding that MAP kinase signaling controls CEP-1–dependent apoptosis by several different pathways that converge on CEP-1. Since apoptosis is also restricted to pachytene stage cells in mammalian germlines, analogous mechanisms regulating p53 family members are likely to be conserved throughout evolution.

## Author Summary

Germ cell apoptosis helps to ensure that only healthy germ cells contribute to the next generation. The *C. elegans* p53 family member CEP-1 plays an important role in inducing apoptosis in damaged germ cells. CEP-1 protein is maximally expressed in late-stage pachytene cells, which are the only cells of the germline that undergo apoptosis. Restricting CEP-1 to late pachytene cells is thought to ensure that apoptosis does not occur in cells at earlier stages of meiosis where meiotic recombination occurs. Through an unbiased genetic screen, we uncovered a role for the Ras/MAP kinase signaling pathway as a novel regulator of DNA damage–induced, CEP-1–dependent apoptosis. We show that the Ras/MAP kinase pathway is required for DNA damage–induced apoptosis by regulating the expression of CEP-1 in late pachytene cells. In addition, MAP kinase signaling might be directly involved in apoptosis induction, as the pathway that is activated in response to IR and MAP kinase directly interacts with CEP-1. We postulate that p53 family members might be regulated by analogous mechanisms in mammals.

## Introduction

The p53 family of transcription factors is conserved throughout animal evolution [1], [2]. In vertebrates the founding member, p53, is a key tumour suppressor and is the most commonly mutated gene in human tumours. Two paralogues, p63 and p73, have diverse roles in development and in responding to cellular stress [3]. Based on sequence similarity it appears that the majority of invertebrate p53 family members are most closely related to mammalian p63 and it has been postulated that an ancient function of the p53 family might be the regulation of germ cell apoptosis [4]. The sole *C. elegans* p53 homologue CEP-1 was implicated in regulating germ cell apoptosis in response to DNA damage and meiotic recombination failure [5], [6]. Interestingly, more recent reports indicate that the TAp63 specific isoform is required to eliminate damaged meiotic germ cells in the mammalian female germline [4].

The *C. elegans* hermaphrodite germline consists of two U-shaped gonads, in which the germ cells are organised in a gradient of maturation. In the distal part of the germline cells proliferate mitotically before entering meiosis in the transition zone. Cells go through the various stages of meiosis as they progress through the germline. Once they have progressed into diplotene and diakenesis they begin oocyte differentiation. Apoptosis is only observed in cells in the late pachytene stage where homologous chromosomes are synapsed and meiotic recombination has been largely completed. A number of different stimuli can induce apoptosis in the germline and all require the same core apoptotic machinery used during *C. elegans* somatic development, including the Bcl-2 family member CED-9, which acts to inhibit the Apaf-1 homologue CED-4, that in turn activates the caspase CED-3 [7]. A low background level of CEP-1 independent death, termed physiological apoptosis, is thought to maintain tissue homeostasis in the germline. In contrast, DNA damage induced apoptosis specifically involves CEP-1 activation by the DNA damage response pathway and the subsequent CEP-1 dependent transcriptional induction of the BH3 only (Bcl-2 homology domain 3) gene *egl-1*. This mechanism is analogous to IR-induced p53 dependent transcriptional induction of NOXA and PUMA in mammals [8], .

The extracellular signal-related kinase ERK is downstream of the MAP kinase signaling pathway that includes the Ras GTPase, and is involved in many aspects of animal development and homeostasis. In *C. elegans*, LET-60 (the Ras homologue), MPK-1 (the ERK homologue), and several other members of the pathway are conserved and are important for many aspects of somatic and germline development and function. During somatic development this pathway is part of an inductive signal required to specify the fate of the vulva [10]. Within the germline Ras/ERK signaling is involved in germline proliferation, meiotic progression, and oocyte maturation and growth [11], and is also required for physiological apoptosis [12], [13]. MAPK phosphatases (MKPs) are important regulators of this signaling pathway, and function by dephosphorylating and deactivating MAPKs. In *C. elegans*, genetic studies have implicated the MKP LIP-1 as an inhibitor of MPK-1 signaling in both the vulva and the germline [14]–[16].

The observation that apoptosis only occurs in cells in the late pachytene stage of meiosis indicates that there must be particular signals or regulatory mechanisms that make only these particular germ cells competent for apoptosis and that prevent apoptosis in all other germ cells. Restricting apoptosis to late pachytene stage cells could prevent the inappropriate loss of cells in both the transition zone and the early pachytene stage where SPO-11 dependent double strand breaks are formed [17]–[19] and meiotic recombination occurs [20], respectively. One way to restrict apoptosis to late pachytene cells is via control of CEP-1 expression in the germline. We previously reported that GLD-1 represses the translation of CEP-1 in early stage meiotic cells and that CEP-1 expression gradually increases as GLD-1 levels decrease in late pachytene [21]. It is likely that further developmental signals are also involved in establishing apoptotic competency, possibly by regulating entry into late pachytene, or regulating the expression of CEP-1 or other apoptotic factors. One such developmental signal is likely to be mediated by MPK-1 activation, which is required for entry into late pachytene [11].

Here we report that MPK-1 signaling regulates CEP-1 dependent, DNA damage induced apoptosis. Using an unbiased genetic screen we found that excessive MAP kinase signaling, conferred by mutations of the MAP kinase phosphatase LIP-1 and by an activating allele of Ras, leads to excessive DNA damage dependent germ cell apoptosis. Conversely, the absence of MPK-1 inhibits DNA damage induced apoptosis. We provide evidence that MPK-1 signaling acts developmentally to regulate apoptosis competency by controlling CEP-1 expression levels in late pachytene cells. Furthermore, we show that MPK-1 signaling is triggered by IR, and that this might directly activate CEP-1.

## Results

### Identification of the *lip-1* MAPK phosphatase as a novel negative regulator of *cep-1*

**Figure 1:**
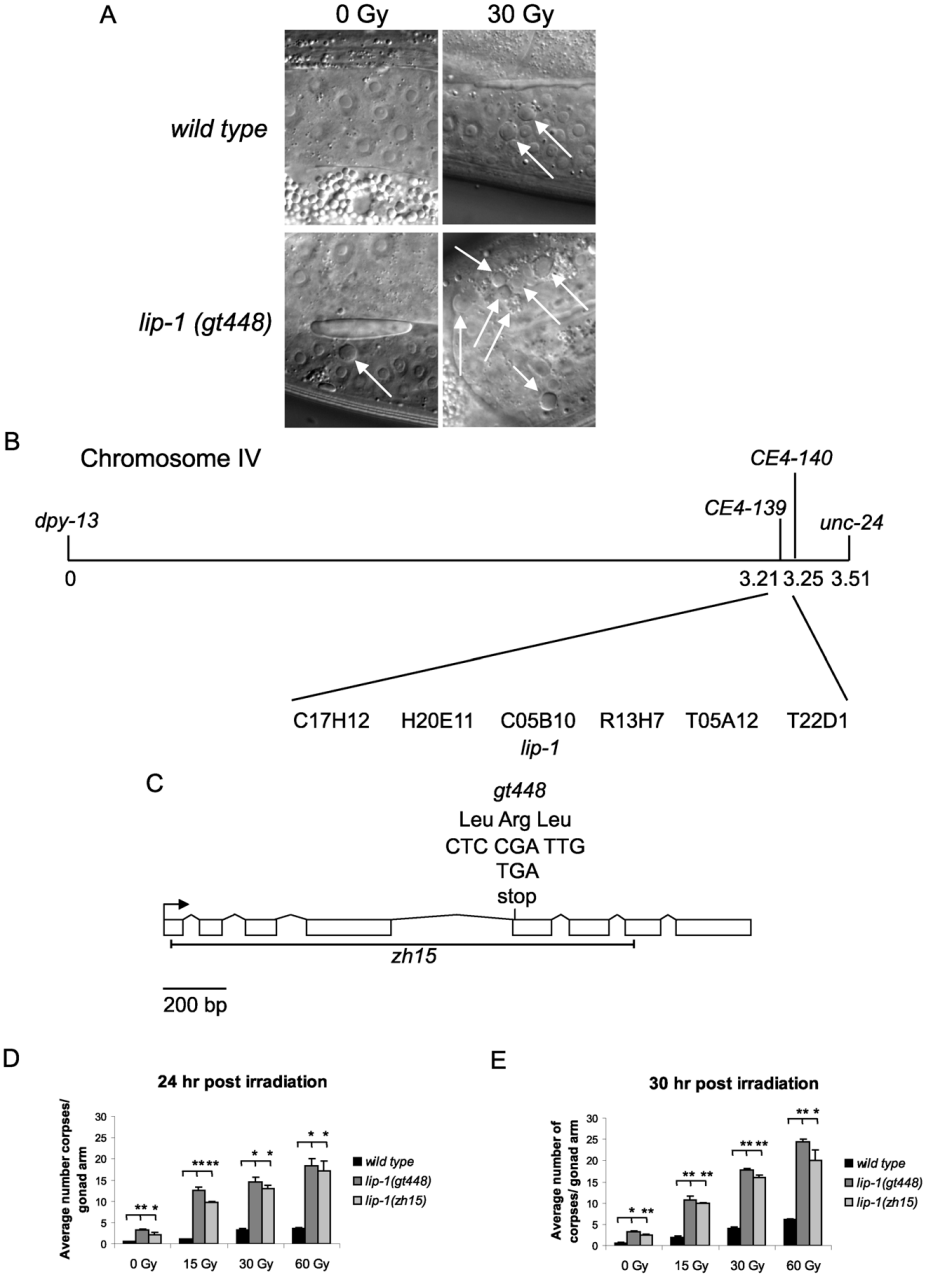
*gt448* is an allele of *lip-1*. (A) *gt448* shows enhanced apoptosis following 30 Gy of ionising irradiation (IR). Wild type (N2) and *gt448* L4 larval stage hermaphrodites were irradiated and observed by Nomarski optics 24 hours later. Apoptotic corpses are indicated by arrows. (B) *gt448* was mapped to chromosome IV between the visible markers *dpy-13* and *unc-24*. Fine mapping using CB4856 positioned *gt488* between the two SNPs *CE4-139* and *CE4-140*. Of the six cosmids in this region, one (C05B10) contained a likely candidate, *lip-1*. (C) Sequencing of the *lip-1* locus identified a C to T change, which introduces a stop codon at amino acid 170. The *zh15* allele is also shown. (D and E) Wild type, *lip-1(zh15)*, and *lip-1(gt448)* hermaphrodite L4 larval stage worms were gamma irradiated at the specified doses and allowed to recover at 20°C for the specified times before apoptotic corpses in the germline were scored by Nomarski optics. Error bars represent the standard error of the mean and the asterisks the p-value for a paired t-test (* p<0.05, ** p<0.01). Animals for each genotype, dose, and time point were assessed in triplicate, scoring a minimum of 15 germlines for each data point.

We previously implicated the translational repressor GLD-1 as a negative regulator of CEP-1 via a genetic screen for mutants showing an enhanced IR induced apoptosis phenotype [21]. To find further negative regulators of *cep-1* we continued this genetic screen and isolated the *gt448* mutant that contains significantly more apoptotic corpses than wild type (N2) worms following low dose IR treatment (30 Gy) (Figure 1A, 1D and 1E). Genetic analyses showed that the increased apoptosis is *cep-1* dependent and is not caused by a DNA repair defect (see below). Mapping with a polymorphic strain, CB4856, positioned *gt448* on linkage group IV, and three-factor mapping located *gt448* between *dpy-13* and *unc-31*. Fine mapping using a *dpy-13 gt448 unc-31* triple mutant strain and CB4856 placed *gt448* between the single nucleotide polymorphisms *CE4-139* and *CE4-140* (Figure 1B). Six cosmids map to this region, one of which contains the *lip-1* (C05B10.1) locus. Sequencing of the coding region of the *lip-1* phosphatase identified a C>T change leading to the conversion of Arg 170 to a stop codon, resulting in a truncated protein lacking the phosphatase catalytic domain (Figure 1C). Non-complementation between *gt448* and the *lip-1 (zh15)* deletion allele [16] confirmed that increased IR induced apoptosis in the *gt448* mutant is due to loss of *lip-1* function (data not shown).

### Enhanced MAPK signaling conferred by loss of *lip-1* or gain of *let-60/*Ras function results in enhanced radiation induced apoptosis

Both *lip-1(gt448)* and *lip-1(zh15)* mutant worms show slightly enhanced levels of apoptosis without irradiation at 20°C (Figure 1D and 1E). However, following low dose IR treatment (15 or 30 Gy) very high levels of CEP-1 dependent apoptosis are observed (Figure 1D and 1E). Previous reports indicate that *lip-1* mutants show enhanced apoptosis when shifted to 25°C (without DNA damage) but no data were shown for growth at 20°C [22]. We also observed increased apoptosis when *lip-1(zh15)* and *lip-1(gt448)* mutants were shifted to 25°C, but this was *cep-1* independent (data not shown).

**Figure 2:**
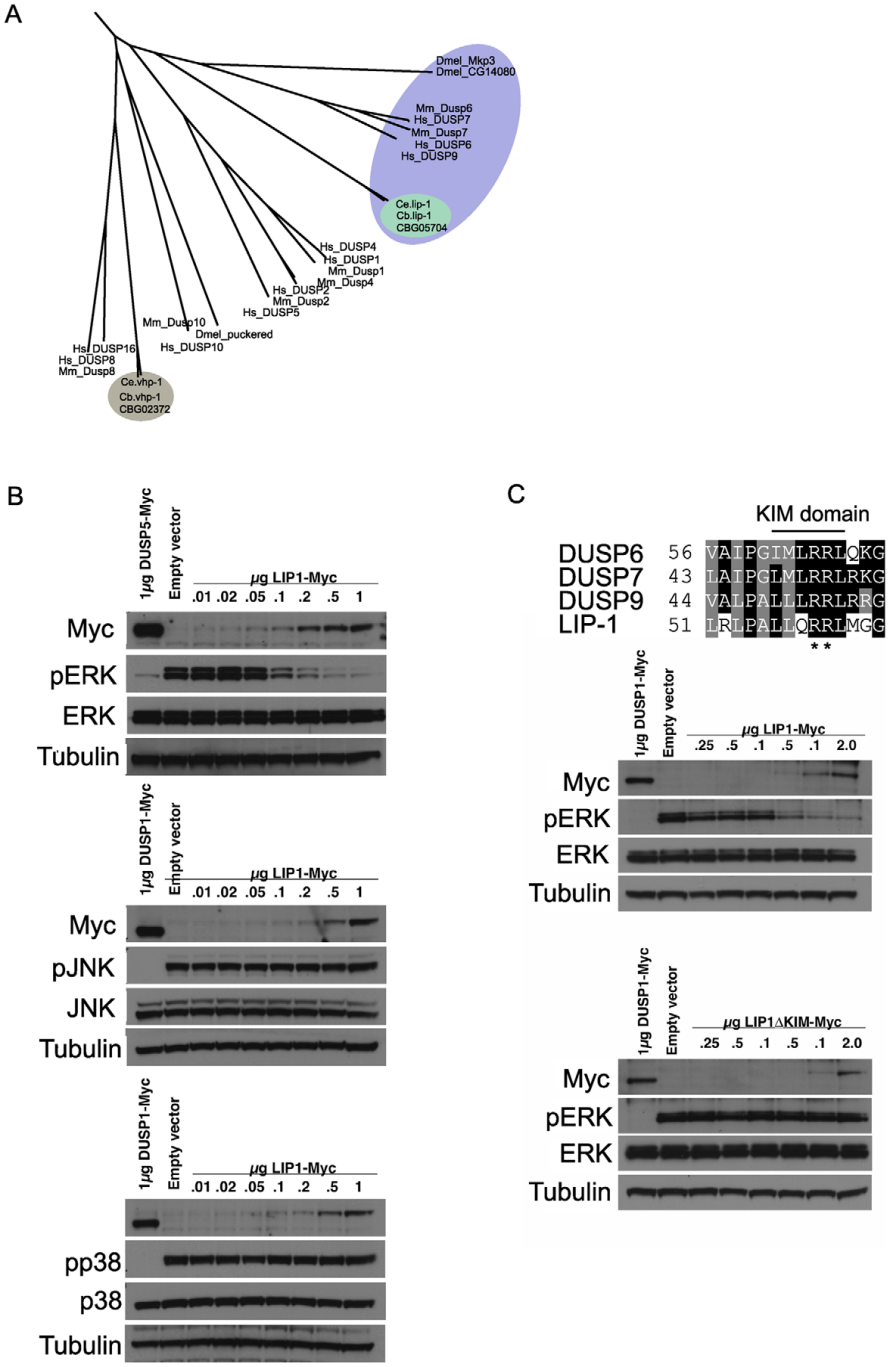
*lip-1* encodes an ERK–specific phosphatase. (A) Phylogenic tree showing the evolutionary relationships of human (Hs), mouse (Mm), *Drosophila* (Dmel), *C. elegans* (Ce) and *C. briggsae* (Cb) MAPK phosphatases. LIP-1 is highlighted by the green oval within the DUSP6/7/9 cluster (blue oval). The other *C. elegans* MAPK phosphatase VHP-1 is highlighted by the grey oval. (B) *In vivo* dephosphorylation assays. Increasing amounts of Myc-tagged LIP-1 was expressed in Cos-1 cells and the phosphorylation of endogenous ERKs 1 and 2 (pERK), JNK (pJNK), or p38 (pp38) in response to either serum stimulation (ERK) or anisomycin (JNK and p38) was monitored by western blotting. Myc-tagged human DUSP5 and DUSP1 were used as positive controls to inactivate ERK and JNK/p38, respectively. (C) Sequence alignment of human DUSP6, DUSP7, DUSP9, and LIP-1, showing the conserved Kinase Interaction Motif (KIM). Increasing amounts of either Myc-tagged wild type or a mutant in which the conserved arginine residues (indicated by asterisks) of the KIM were mutated to alanine were expressed in Cos-1 cells and the phosphorylation of ERK1/2 was assessed. Tagged human DUSP1 was used as a positive control.

The LIP-1 protein has been reported to be a MPK-1 phosphatase, based on its sequence homology with mammalian MAPK phosphatases and genetic analyses that implicated it as an inhibitor of *mpk-1* [14]–[16]. To ascertain that the excessive apoptosis phenotype of *lip-1* mutants is indeed linked to MPK-1 activation we first wished to confirm that LIP-1 acts as an MPK-1 phosphatase. We thus carefully assessed the phylogenetic relationship between LIP-1 and other known dual specificity protein phosphatases, including MAPK phosphatases, and tested directly which MAPK family members are inactivated by LIP-1. LIP-1 clusters with the mammalian ERK specific phosphatases DUSP6, 7, and 9 and *Drosophila* Mkp3 (Figure 2A, reviewed in [23]), whereas the other *C. elegans* MAPK phosphatase orthologue VHP-1 (F08B1.1), clusters with DUSP 16 and 8, both of which show substrate specificity for the JNK and p38 MAPKs (Figure 2A, reviewed in [23]). In agreement with our phylogenetic analysis, and extending the *in vitro* study performed by Mizuno *et al.*, which indicated that LIP-1 shows specificity towards human ERK *in vitro* [24], our *in vivo* analysis in Cos-1 cells established that the expression of epitope-tagged LIP-1 leads to the inactivation of endogenous ERK1 and ERK2 but not of either the p38 or JNK MAPKs (Figure 2B). Furthermore, LIP-1 activity towards ERK is absolutely dependent on the integrity of a conserved Kinase Interaction Motif (KIM) located within the non-catalytic amino-terminal domain of LIP-1 (Figure 2C). LIP-1 thus shares a common mechanism of substrate recognition and catalysis with the mammalian ERK-specific phosphatase DUSP6/MKP-3, and likely acts to specifically inhibit the *C. elegans* ERK MPK-1 [25].

**Figure 3:**
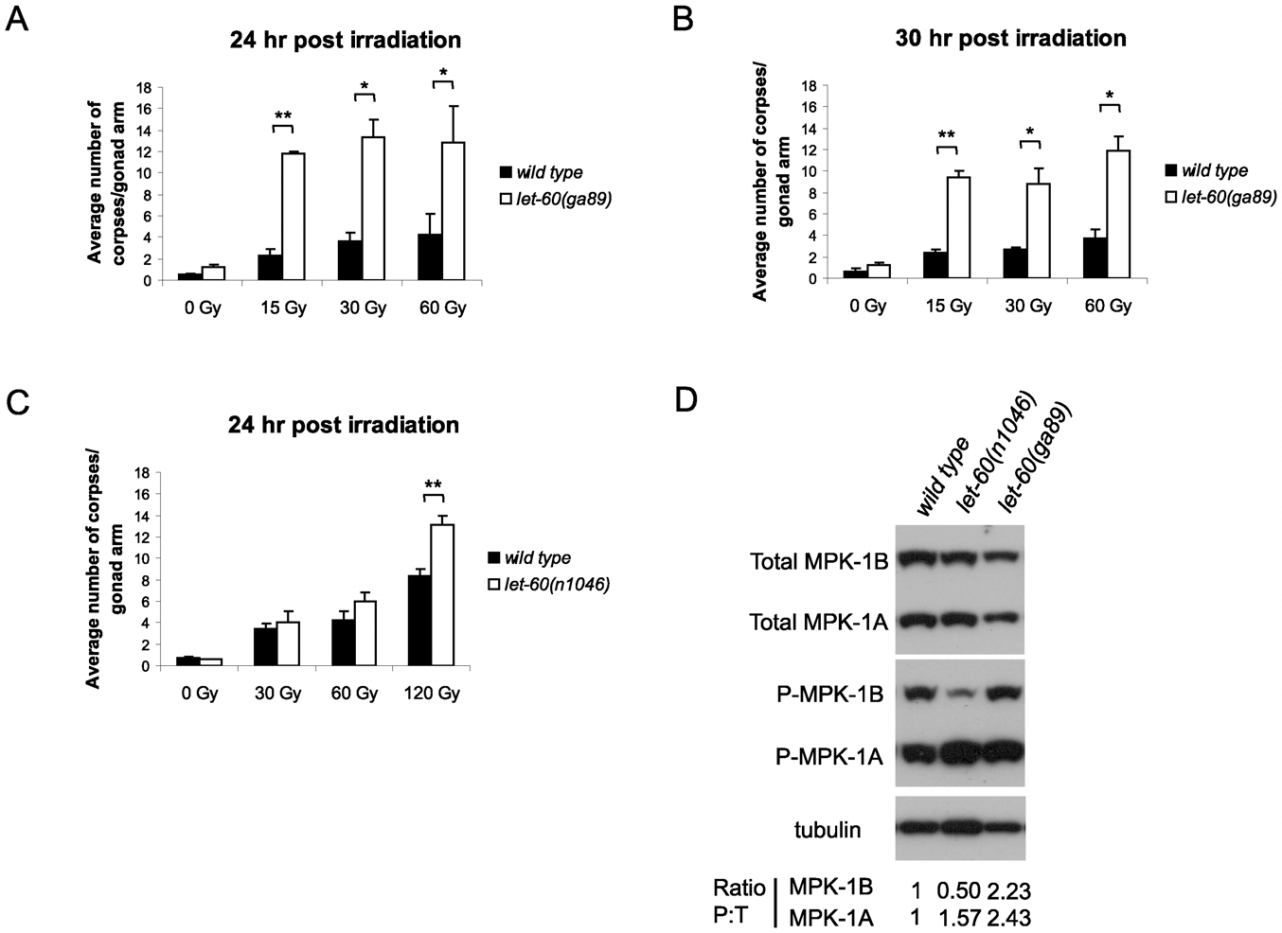
*let-60(ga89)* mutants show enhanced apoptosis following irradiation. (A and B) Wild type and *let-60(ga89)* and (C) wild type and *let-60(n1046)* worms were irradiated and allowed to recover at 20°C, and apoptotic corpses were scored after the stated time points. The scoring of apoptotic corpses was performed as in Figure 1. (D) Activated (phosphorylated) MPK-1 levels differ between *let-60(ga89)* and *let-60(n1046)* worms. Protein was extracted from young adult worms (24 hours post L4 larval stage) and equal amounts were loaded onto SDS-PAGE gels. Activated MPK-1 was detected by an anti-P-ERK antibody, total MPK-1 by an anti-ERK antibody, and α-tubulin was used to control for loading. The ratio of phosphorylated MPK-1 to total MPK-1 (Ratio P∶T, normalised against wild type) is shown for the two isoforms.

Having demonstrated that LIP-1 directly antagonizes ERK, we next tested whether activation of MPK-1 by a gain of function *let-60*/Ras allele results in increased apoptosis. At 20°C (the temperature used in these experiments) *let-60(ga89)* acts as a weak gain of function allele, while at 25°C it acts as a strong gain of function allele showing both somatic and germline phenotypes [26]. Similar to loss of *lip-1*, *let-60(ga89)* worms raised at 20°C show greatly elevated levels of IR induced apoptosis (Figure 3A and 3B). Interestingly, worms mutant for *let-60(n1046)*, another gain of function allele, do not show enhanced apoptosis following low doses of IR but do show elevated apoptosis after higher levels (120 Gy) (Figure 3C). *let-60(n1046)* is a constitutive mutant allele reported to lead to excessive vulva formation but which has no effect on germline development [11].

Since we observed a difference in apoptosis induction in the two *let-60* gain of function alleles we examined MPK-1 protein and phosphorylation levels in these mutants by immunoblotting with antibodies recognising mammalian ERK and phosphorylated ERK that cross react with MPK-1 [11], [27], [28]. MPK-1 is expressed as two isoforms that result from alternative splicing [29], [30]: MPK-1A is the smaller isoform that appears to be predominantly somatic, whereas MPK-1B is larger and is expressed only in the germline [31]. *let-60(ga89)* mutants show reduced levels of total MPK-1A and B (Figure 3D). Despite the reduction in total protein levels, both MPK-1 isoforms are hyperphosphorylated in this mutant (Figure 3D, the ratio of phosphorylated to total protein is ∼2.4 times greater than wild type for MPK-1A and ∼2.2 times for MPK-1B). On the other hand, *let-60(n1046)* shows reduced phosphorylation of MPK-1B (only 0.5 times that of wild type) but hyperphosphorylation of MPK-1A (∼1.6 times) and no change in total protein levels (Figure 3D). Thus, hyperphosphorylation of the MPK-1B germline isoform correlates with the hyperinduction of apoptosis following low dose irradiation. Our findings are consistent with a previous report indicating that the pattern of germline MPK-1 phosphorylation varies in *let-60(ga89)* and *let-60(n1046)* mutants [11].

### Elevated damage-induced apoptosis in *lip-1(lf)* and *let-60(ga89)* mutants is dependent on the *cep-1/p53* pathway

**Figure 4:**
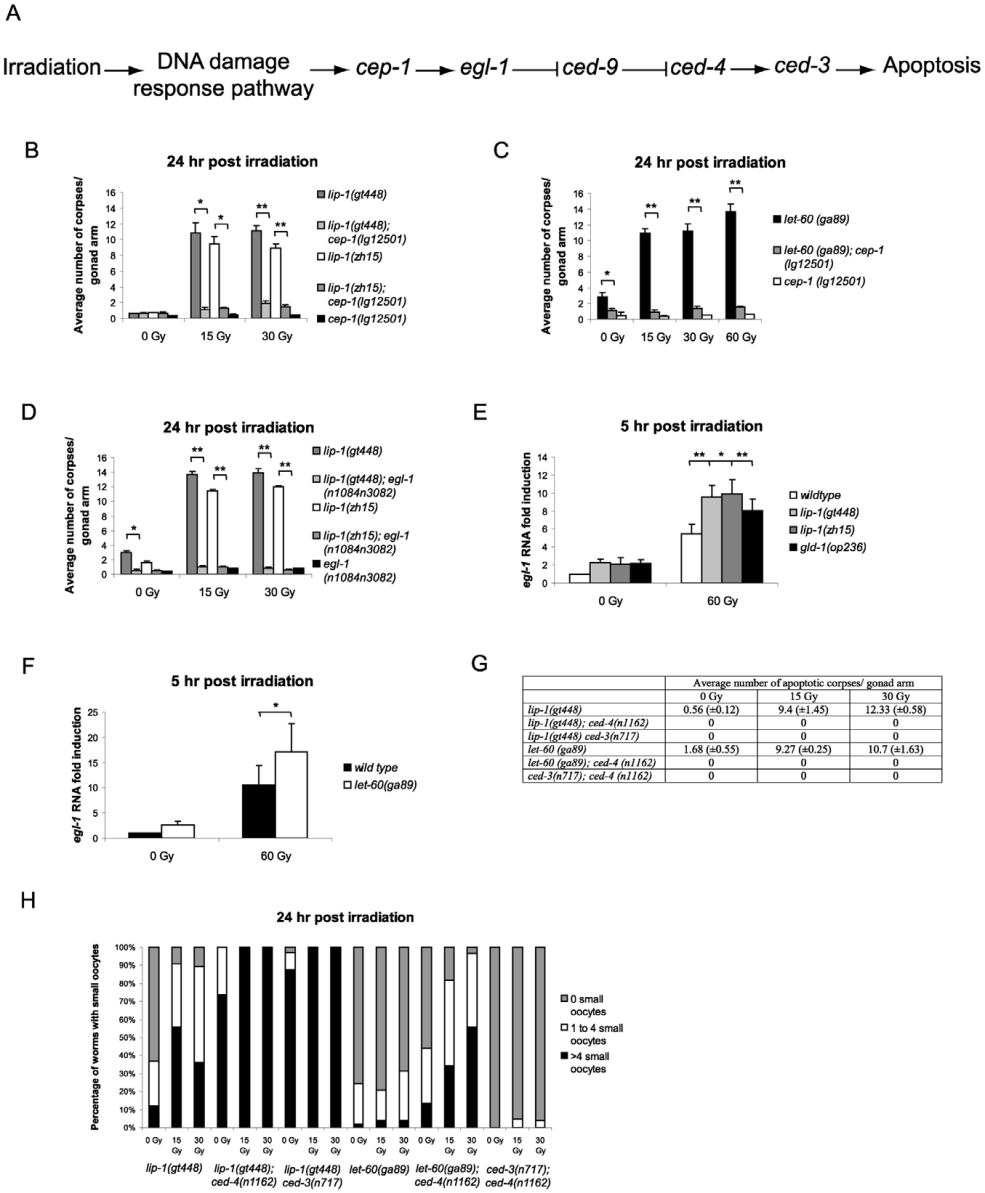
Epistasis between *lip-1(lf)* or *let-60(ga89)* and members of the *cep-1* dependent apoptotic pathway. (A) Scheme of the *cep-1* dependent apoptotic pathway. The enhanced apoptosis following irradiation observed in *lip-1(lf)* and *let-60(ga89)* mutants is dependent on *cep-1* (B and C) and *egl-1* (D). In *lip-1(lf)* (E) and *let-60(ga89)* (F) worms *egl-1* transcription is enhanced, as measured by quantitative real time PCR. Young adult worms were irradiated with the specified dose and mRNA was isolated 5 hours later. mRNA levels for each sample were normalised to γ-tubulin and the fold induction was calculated relative to the levels in wild type untreated worms. Biological experiments were done in triplicate (*lip-1*) or quadruplicate (*let-60(ga89)*). Error bars represent the standard error of the mean and asterisks represent the p-value for a paired t-test (* p<0.05, ** p<0.01). (G) Loss of *ced-3* or *ced-4* suppresses the apoptosis observed in *lip-1(gt448)* and *let-60(ga89)* mutants. (H) The small oocyte phenotype of *lip-1(gt448)* or *let-60(ga89)* mutants is enhanced by concomitant loss of *ced-3* or *ced-4*. Worms were scored as containing no small oocytes (0), four or less (1 to 4), or greater than four (>4). The scoring of apoptotic corpses was performed as in Figure 1.

In the *C. elegans* germline IR induced apoptosis is mediated by *cep-1* (*p53* homologue) dependent transcription of the BH3 only protein *egl-1* (Figure 4A) [21]. In contrast, physiological apoptosis does not require either *cep-1* or *egl-1* [5], [12]. We were therefore interested in determining whether the increased IR dependent apoptosis observed in *lip-1(lf)* and *let-60(ga89)* mutants was mediated by the *cep-1* pathway. For this, we generated double mutant strains containing combinations of either the *lip-1(lf)* or *let-60(ga89)* alleles with mutant alleles of apoptotic pathway components. We found that the enhanced apoptosis following irradiation observed in the *lip-1(lf)* and *let-60(ga89)* mutants is suppressed by the absence of *cep-1* (Figure 4B and 4C) and *egl-1* (Figure 4D) function. To test whether *cep-1* dependent *egl-1* transcription is enhanced in *lip-1(lf)* and *let-60(ga89)* mutants, we measured *egl-1* RNA levels by quantitative PCR. In both *lip-1(lf)* and *let-60(ga89)* mutants there is increased IR-induced *egl-1* transcription (Figure 4E and 4F, [21]). The *gld-1(op236)* mutant, which we have previously shown to cause excessive *egl-1* transcription, was used as a positive control [21]. All germline apoptosis requires the Apaf1 homologue, *ced-4*, and the caspase *ced-3* (Figure 4A). Therefore, as expected, in the absence of either *ced-4* or *ced-3* function no apoptosis is observed in *lip-1(gt448)* or *let-60(ga89)* mutants (Figure 4G). Interestingly, however, loss of either *ced-4* or *ced-3* enhances the small oocyte phenotype of *lip-1(gt448)* and *let-60(ga89)* mutants (Figure 4H). Old *ced-3* and *ced-4* worms have been reported to lay small oocytes of poor quality with the quality decreasing as the worms age, indicating that germ cell apoptosis is necessary to contribute to oocyte growth and viability by allocating scarce resources to the developing oocyte [12], [32]. Our finding that loss of both *lip-1* and either *ced-3* or *ced-4* results in a much larger number of small oocytes indicates that both proper levels of apoptosis and MPK-1 activation independently regulate oocyte growth.

### Enhanced damage-induced apoptosis observed in *lip-1* and *let-60* mutants is not due to a DNA repair defect

Defective DNA repair results in an enhanced apoptotic phenotype following IR due to the persistence of DNA double strand breaks that continually activate damage response pathways. To ensure that the enhanced apoptosis in *lip-1(lf)* and *let-60(ga89)* mutants is not due to a defect in DNA repair following IR, we examined the survival rate of progeny laid by irradiated mothers. Mutants that are defective in DNA repair (e.g. *mrt-2(e2663)* [33]) show a marked reduction in progeny survival rate following IR (see Table 1) due to the inheritance of broken chromosomes from their mothers. Unlike *mrt-2(e2663)* mutant worms, the survival rate of progeny arising from normal (i.e. not small) eggs from *lip-1* and *let-60* mutant mothers is not significantly different from that of wild type worms (Table 1). As reported previously [15] and confirmed above (Figure 4H), *lip-1* mutant worms also lay small eggs and unfertilised oocytes (that can be identified by their flattened and brown appearance due to a lack of an eggshell). We also observed this phenotype in *let-60(ga89)* but not *let-60(n1046)* worms (Table 1). The rate at which these abnormal eggs/oocytes were laid was not changed by irradiation. However, the survival rates of progeny from small eggs did decrease in *lip-1(lf)* and *let-60 (ga89)* mutants following irradiation. Nevertheless, the extent of survival reduction was less than observed for *mrt-2(e2663)*. Since these eggs are already abnormal the cause of the change in their survival rate is unclear, but is unlikely to be related to a reduced DNA repair capacity. In summary, our data show that the enhanced apoptosis observed in *lip-1(lf)* and *let-60(ga89)* worms is not due to a DNA repair defect as the survival rate of progeny derived from normal sized eggs is not affected by irradiation.

**Table 1:** *lip-1(lf)* and *let-60(gf)* mutants do not show radiation sensitivity.

	0 Gy						30 Gy					
Genotype	Normal eggs laid^a^	% survival	Small eggs laid^a^^,^ b	% survival	Oocytes laid^a^^,^ c	% survival	Normal eggs laid^a^	% survival	Small eggs laid^a^^,^ b	% survival	Oocytes laid^a^^,^ c	% survival
*N2*	3.67 (±0.37)	99.43 (±0.29)	0	-	0	-	3.94 (±0.25)	97.71 (±0.85)	0	-	0	-
*mrt-2*	4.56 (±0.36)	96.95 (±0.96)	0	-	0	-	4.31 (±0.08)	51.29 (±6.08)	0.32 (±0.07)	13.04 (±11.76)	0	-
*lip-1(gt448)*	2.60 (±0.27)	99.20 (±0.9)	1.21 (±0.20)	55.75 (±3.16)	0.09 (±0.09)	0	1.26 (±0.24)	94.51 (±2.68)	0.99 (±0.13)	22.54 (±6.79)	0.01 (±0.01)	0
*lip-1(zh15)*	2.60 (±0.10)	99.20 (±0.47)	0.81 (±0.42)	62.07 (±14.71)	0	-	0.37 (±0.07)	96.23 (±2.40)	0.81 (±0.14)	41.38 (±11.26)	0.01 (±0.01)	0
*bcIs39*	3.76 (±0.35)	100.00 (±0)	0.01 (±0.01)	0	0	-	3.03 (±0.36)	92.91 (±3.32)	0	-	0	-
*let-60(ga89)*; *bcIs39*	3.99 (±0.07)	99.48 (±0.29)	0.27 (±0.04)	51.28 (±2.80)	0.06 (±0.03)	0	2.39 (±0.09)	88.95 (±4.68)	0.67 (±0.11)	64.95 (±20.16)	0	-
*let-60(n1046)*; *bcIs39*	3.74 (±0.21)	98.70 (±0.41)	0	-	0	-	3.40 (±0.21)	95.10 (±2.18)	0	-	0	-

L4 larval stage worms were treated with either 0 or 30 Gy of irradiation and allowed to recover for 24 hours. Worms were then transferred to fresh plates in triplicate and allowed to lay eggs for 24 hours. The number of eggs laid was counted immediately after removing the adult worms and the number remaining was counted 24 hours later (which is sufficient time for L1 larvae to hatch).

a The number of eggs laid is the average number per hour (± SEM).

b Small eggs were defined as those that were round (instead of oval) and half the size or smaller than normal eggs.

c Oocytes were identified by their brown colour and flattened appearance.

### Enhanced apoptosis observed in *lip-1* and *let-60* mutants is due to enhanced MPK-1 activity

**Figure 5:**
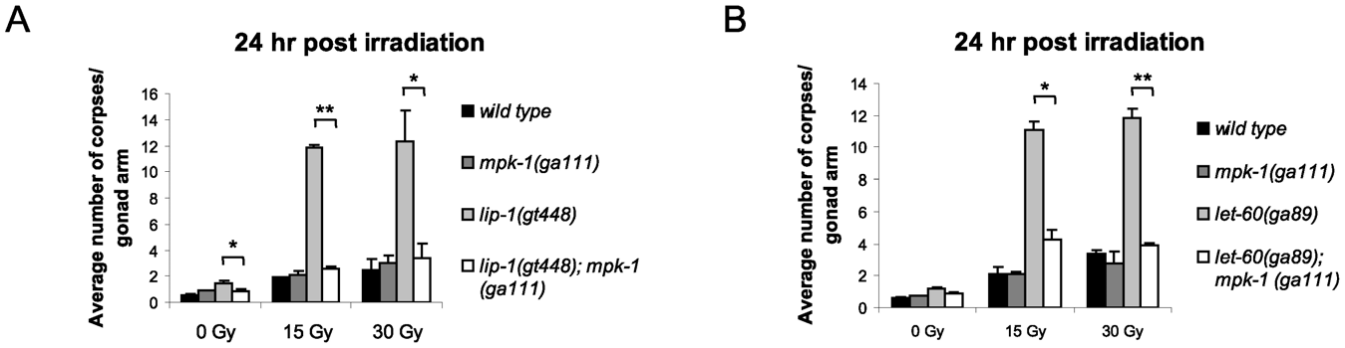
The enhanced apoptosis in *lip-1(gt448)* and *let-60(ga89)* mutant worms is due to enhanced MPK-1 activity. (A and B) The increased apoptosis observed in *lip-1(gt448)* and *let-60(ga89)* worms is suppressed by *mpk-1(ga111ts)* when raised at 20°C (permissive temperature). The scoring of apoptotic corpses was performed as in Figure 1.

As both loss of *lip-1* and gain of *let-60* activity results in increased MPK-1 signaling, we tested whether the enhanced apoptosis observed in these mutants was dependent on enhanced MPK-1 activity. To do this, we generated double mutants of either the *lip-1(lf)* or *let-60(ga89)* alleles with the *mpk-1(ga111ts*) allele. *ga111ts* is a weak loss of function allele containing a mutation in the MEK binding site, which likely reduces the rate at which MPK-1 is phosphorylated and activated, and which at the restrictive temperature of 25°C results in an incomplete pachytene arrest phenotype [30]. In contrast, at the permissive temperature of 20°C *mpk-1(ga111ts)* worms appear wild type [30] and have normal levels of IR induced apoptosis (Figure 5A and 5B). Interestingly, at 20°C the *mpk-1(ga111ts)* allele could fully suppress the enhanced IR induced apoptosis observed in *lip-1(gt448)* and *let-60(ga89)* worms (Figure 5A and 5B), indicating that partially functional MPK-1 is sufficient to suppress the enhanced apoptosis phenotype. This finding demonstrates that elevated MPK-1 activity is required for the enhanced apoptosis induction observed following irradiation in the *lip-1(lf)* and *let-60(ga89)* mutants.

### MPK-1 signaling affects CEP-1 protein expression in the germline

**Figure 6:**
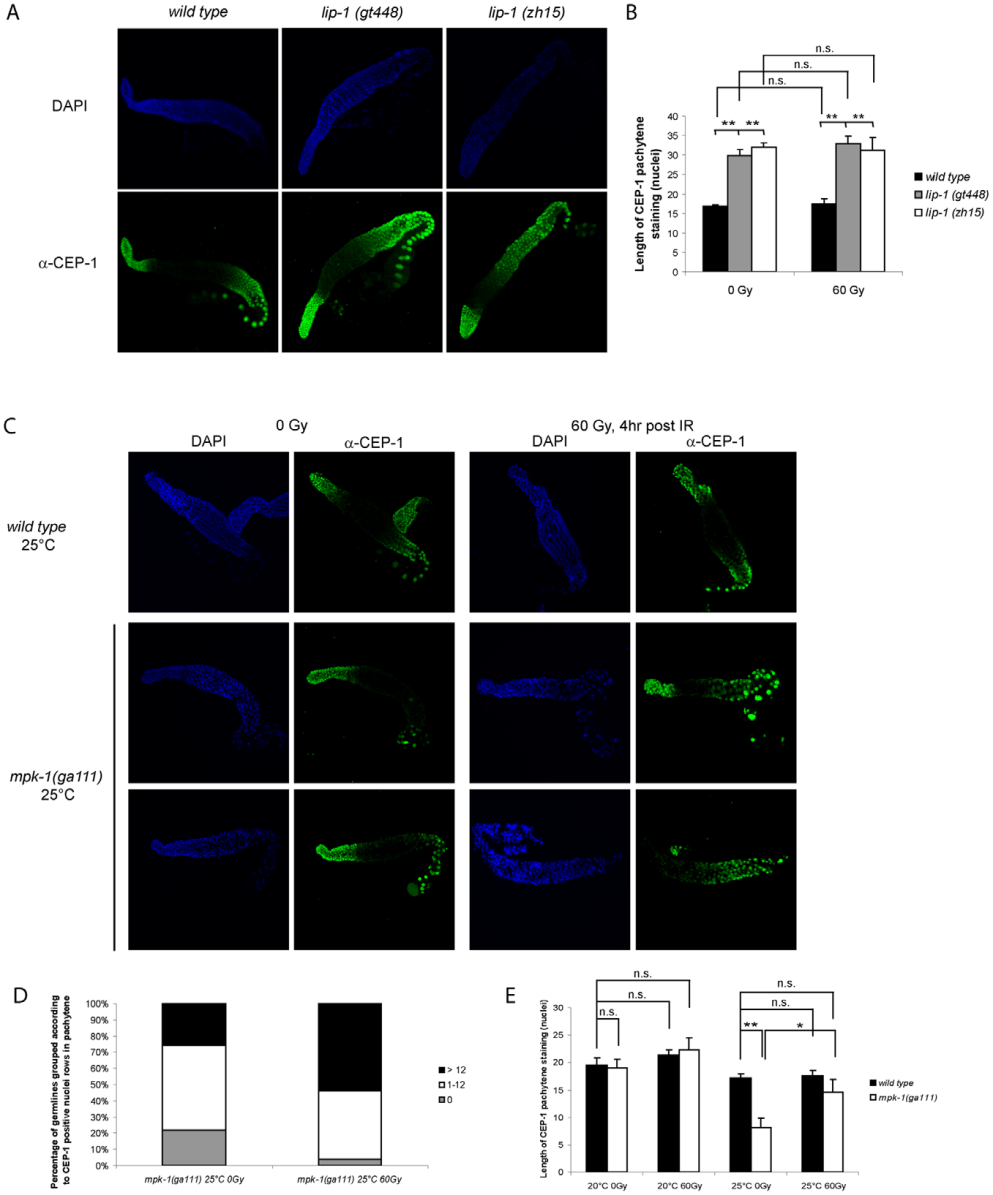
CEP-1 germline expression is affected by MPK-1 signaling. (A and B) CEP-1 protein expression is increased in the germline of *lip-1* mutants. (A) Germlines from young adult hermaphrodites were dissected and processed for immunofluorescence using an anti-CEP-1 antibody (green). DNA was visualised by DAPI staining (blue). All germlines are orientated the same way with the distal end to the left and the proximal end to the right. (B) For statistical analysis the length of CEP-1 pachytene staining was quantified in germlines of each genotype treated with either 0 Gy or 60 Gy and dissected 4 hours later. Quantification was performed by scoring the number of rows of germ cell nuclei reaching from the pachytene/diplotene border to the most distal point in pachytene in which immunofluorescence was first discernable. At least five germlines were counted for each genotype/treatment. The error bars represent the standard error of the mean and the asterisks the p-value for an unpaired t-test (* p<0.05, ** p<0.01, ‘n.s.’ stands for not significant). (C, D and E) CEP-1 protein is reduced in the germline of *mpk-1(ga111ts)* mutants raised at the restrictive temperature of 25°C and is increased upon IR treatment. Worms raised at 20°C or 25°C were treated and germlines were dissected and processed for immunofluorescence as in (A). (D) More *mpk-1(ga111ts)* germlines at 25°C show CEP-1 expression following IR. The number of germlines with pachytene CEP-1 expression were counted and put into three groups: those with expression in more than 12 rows of nuclei, those with expression in 1 to 12 rows, and those with no expression in pachytene stage cells. CEP-1 expression in more than 12 rows implies wild type expression as 13 rows was observed to be the smallest extent of expression in wild type germlines. (E) Average levels of CEP-1 expression are increased in *mpk-1(ga111ts)* germlines. At least three germlines were counted for each genotype/treatment and the data are presented as in (B).

We had previously shown that CEP-1 protein expression occurs in a distinct pattern within the germline [21]. CEP-1 is expressed distally in the mitotic zone and proximally in late pachytene, diplotene, and diakinesis stage meiotic germ cells (Figure 6A). CEP-1 expression in the proximal region of the germline is regulated by the translational repressor GLD-1 [21]. Since MPK-1 signaling is required for progression into late pachytene [11], we wondered if the enhanced CEP-1 dependent apoptosis observed in the *lip-1(lf)* mutants could involve increased expression of CEP-1 in the proximal germline. We therefore examined the expression pattern of CEP-1 in dissected germlines by immunofluorescence using an anti-CEP-1 antibody [21] that shows specificity for CEP-1 (Figure S1). Both *lip-1* loss of function mutants show increased overall CEP-1 expression, with CEP-1 being detected at earlier stages of pachytene compared to wild type (Figure 6A). Quantification of the range of CEP-1 expression, done by measuring the number of rows of nuclei from the beginning of a discernable CEP-1 fluorescent signal in pachytene to the first diplotene nuclei, confirms this finding (Figure 6A, right panel). The pattern and extent of CEP-1 expression is not affected by irradiation in any of the three genotypes examined (data not shown).

To further explore the relationship between MPK-1 signaling and CEP-1 germline protein levels we examined CEP-1 expression in *mpk-1(ga111ts)* mutants. As expected, based on the apoptotic phenotype of these mutants (Figure 5), the pattern of CEP-1 expression was indistinguishable from wild type worms raised at 20°C (data not shown, but for statistical analysis see Figure 6D). However, when raised at the restrictive temperature of 25°C the *mpk-1(ga111ts)* mutant germlines clearly have less CEP-1 in the pachytene region (Figure 6B, 6C and 6D). Representative images are shown to illustrate the patterns of CEP-1 expression observed in this mutant. Occasionally a normal looking germline with normal CEP-1 expression was observed. However, most germlines had either no pachytene but some diplotene expression or a patchy/small amount of pachytene expression. In summary, loss of *lip-1* and loss of *mpk-1* activities have opposing effects on CEP-1 germline expression. We noted that while IR has no effect on CEP-1 expression in wild type germlines (Figure 6A, 6B and 6D and [21]), *mpk-1(ga111ts)* germlines show rescue of CEP-1 expression: more germlines show a wild type or overexpression pattern (>12 nuclei rows) or partial rescue (0–12 nuclei rows) (Figure 6C) and the average extent of CEP-1 expression (as measured by nuclei rows) approached wild type levels (Figure 6D) (see below).

### MPK-1 signaling controls CEP-1 levels through GLD-1–dependent and –independent mechanisms

**Figure 7:**
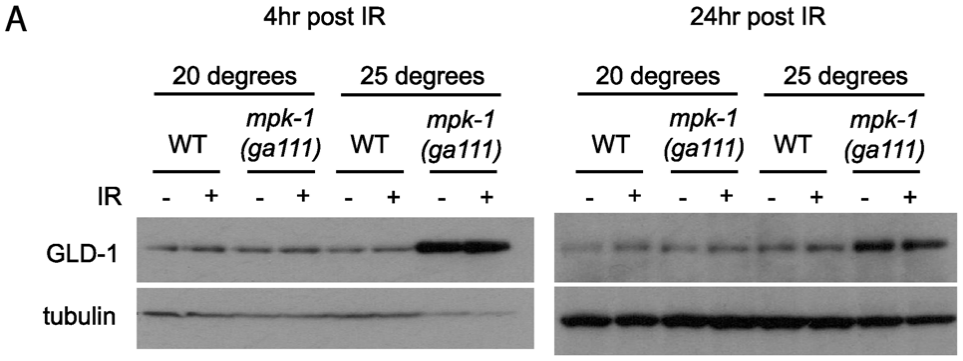
GLD-1 levels are affected by MPK-1 signaling.

Our data clearly show that MPK-1 signaling influences CEP-1 expression in the pachytene region of the germline. We previously reported that the translational repressor GLD-1 regulates CEP-1 expression in late pachytene [21], and it has been reported that GLD-1 protein does not disappear in the proximal region of the germline in *mpk-1* mutants [11], raising the possibility that control of CEP-1 expression by MPK-1 signaling is mediated by GLD-1. To test whether GLD-1 expression may be regulated by MPK-1 signaling we generated GLD-1 specific antibodies (Figure S2) and examined GLD-1 protein levels by immunoblotting. In accordance with a previous published report [11], *mpk-1(ga111ts)* mutants raised at 25°C show increased levels of GLD-1, whereas at the permissive temperature of 20°C GLD-1 levels are the same as wild type (Figure 7A). These findings indicate that GLD-1 levels are influenced by MPK-1 signaling and that this may form part of the mechanism controlling CEP-1 levels.

Since GLD-1 levels and CEP-1 germline expression are both affected by MPK-1 signaling we asked whether MPK-1 regulation of CEP-1 protein levels is mediated by GLD-1. As described above, we have observed that CEP-1 levels are low in *mpk-1(ga111ts)* mutants raised at 25°C and that IR treatment can restore CEP-1 levels to those of wild type (Figure 6). If CEP-1 expression is solely mediated by GLD-1 then we would expect that irradiation should reduce the heightened GLD-1 levels observed in the *mpk-1(ga111ts)* mutant. Against expectation we observe that GLD-1 levels remain unchanged in the *mpk-1(ga111ts)* mutants upon IR, even 24 hours post treatment (Figure 7A). These findings indicate that even though the reduced CEP-1 expression of *mpk-1(ga111ts)* mutants raised at 25°C can be rescued by IR, GLD-1 levels are not altered, and suggest that MPK-1 regulation of CEP-1 expression is not solely mediated by GLD-1.

### MPK-1 is activated in the germline by ionising irradiation

Our finding that radiation rescues CEP-1 expression levels in *mpk-1(ga111ts)* mutants independent of changes in GLD-1 protein levels led us to examine the effect of IR on MPK-1 activity. Since *mpk-1(ga111ts)* is a partial loss of function allele, even at 25°C, it appeared possible that IR activates MAPK signaling, leading to more CEP-1 expression. If this hypothesis is correct IR might be able to restore *mpk-1(ga111ts)* activity, potentially leading to a rescue of the developmental germline defects associated with *mpk-1(ga111ts)* mutants.

**Figure 8:**
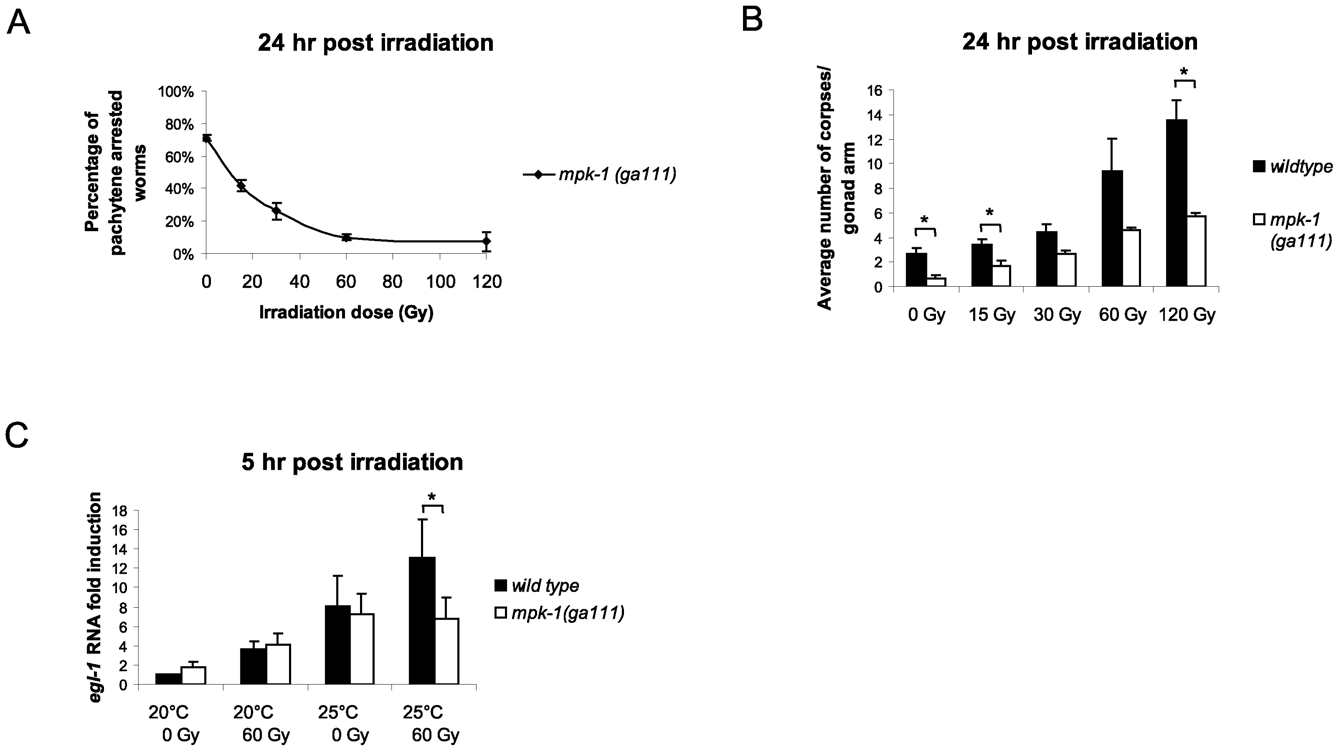
MPK-1 is activated following irradiation and is required for IR–induced *egl-1* transcription. (A) *mpk-1(ga111ts)* worms were raised at 25°C, which results in a pachytene arrest phenotype with 70% penetrance. *mpk-1(ga111ts)* L4 hermaphrodites were irradiated with the specified dose and allowed to recover at 25°C for 24 hours. The percentage of worms displaying a pachytene arrested phenotype is shown. (B) These same worms were also examined for the number of apoptotic corpses present as described in Figure 1. (C) Loss of *mpk-1* results in reduced *egl-1* transcription induction following IR. Wild type and *mpk-1(ga111ts)* worms were raised at 20°C (permissive temperature) and 25°C (restrictive temperature) and gamma irradiated with the specified dose as young adults (24 hour post L4 larval stage). mRNA was harvested 5 hours later, and the *egl-1* transcription assay was performed with quadruple biological replicates as in Figure 4E.

To examine the effects that IR has on MPK-1 signaling we analysed *mpk-1(ga111ts)* mutant worms that had been raised at 25°C. Unirradiated worms show an incomplete pachytene arrest phenotype with approximately 70% of *mpk-1 (ga111ts)* mutant worms containing germlines arrested at the pachytene stage with no oocytes or embryos [11], [30] (Figure 8A). However, if *mpk-1 (ga111ts)* worms are irradiated and allowed to recover at 25°C, a dose dependent rescue of the pachytene arrest is observed as the proportion of intact, fully developed germlines increases (Figure 8A). These data indicate that IR activates MPK-1 signaling. Another allele of *mpk-1*, *oz140*, is functionally null and shows a fully penetrant pachytene arrest that is not temperature sensitive [30]. We did not observe a rescue of the pachytene arrest in irradiated *mpk-1(oz140)* mutant worms (data not shown), indicating that the rescue observed in the *ga111ts* worms is due to increased MPK-1 activity rather than through bypassing the requirement for MPK-1 in pachytene progression.

Since IR can rescue both the pachytene arrest phenotype and CEP-1 expression levels of *mpk-1(ga111ts)* mutants we were interested in measuring the apoptotic response of these worms to assess whether pachytene progression and CEP-1 expression was sufficient for a normal apoptotic response. When the irradiated pachytene-rescued worms were examined for apoptosis induction they were found to contain fewer corpses than wild type worms (Figure 8B). Even at the high dose of 120 Gy, where almost 100% of the mutant worms exhibit normal germlines, the level of apoptosis was greatly reduced compared to wild type, indicating that full MPK-1 activation is needed for apoptosis induction. The inability to induce wild type levels of apoptosis in the *mpk-1(ga111ts)* worms was not due to hypoproliferation of the germline as dissected germlines from *mpk-1(ga111ts)* mutant worms were of the same size as those of wild type, both with and without irradiation (Figure S3A, the smaller germline observed in the wild type after 120 Gy of irradiation is likely due to the high levels of apoptosis induced under these conditions) and there is no difference in the number of phospho-histone H3 positive M phase cells [34], [35] in the mitotic zone of *mpk-1(ga111)* germlines compared to wild type (Figure S3B). Since we observed reduced apoptosis induction in irradiated *mpk-1(ga111ts)* worms despite almost normal levels of CEP-1 expression, we tested whether MPK-1 signaling plays a direct role in apoptosis induction following IR. To do this, we examined *egl-1* transcriptional induction in wild type and *mpk-1(ga111ts)* worms raised at both 20°C and 25°C treated with either 0 Gy or 60 Gy. At 20°C *mpk-1(ga111ts)* worms show normal apoptosis induction following IR treatment (see Figure 5) correlating with wild type levels of *egl-1* induction with and without irradiation treatment (Figure 8C). When raised at 25°C unirradiated *ga111ts* mutant worms have equivalent levels of *egl-1* mRNA to wild type worms, but greatly reduced levels of *egl-1* transcriptional induction following irradiation (Figure 8C), indicating that high MPK-1 activity is required for *egl-1* transcriptional induction by CEP-1 following irradiation. Thus, (I) high levels of MPK-1 signaling are required to trigger CEP-1 dependent *egl-1* transcription upon IR, and the restoration of wild type levels of CEP-1 in *mpk-1(ga111ts)* worms rescued by ionising irradiation is not sufficient to trigger apoptosis, and (II) MPK-1 signaling plays an additional role in activating CEP-1 dependent apoptosis. In summary, our findings imply that the reduced apoptosis observed is due neither solely to an inability to enter into late pachytene where apoptosis occurs nor to defects in germline proliferation. Rather MPK-1 plays two roles: one in pachytene progression (and CEP-1 expression) and another in DNA damage dependent apoptosis induction.

**Figure 9:**
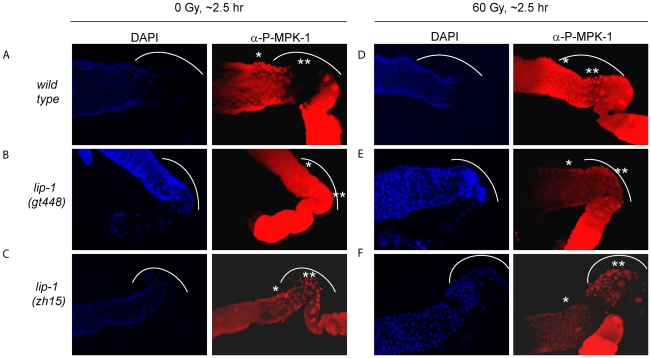
MPK-1 phosphorylation is induced in late pachytene and early diplotene in wild-type germlines following irradiation. (A and D) Wild type, (B and E) *lip-1(gt448)*, and (C and F) *lip-1(zh15)* worms (24 hours post L4 larval stage) were treated with 0 or 60 Gy of gamma irradiation and allowed to recover for two hours. Germlines were then dissected and processed for immunofluorescence using an anti-P-MPK-1 antibody (red). Nuclei were stained with DAPI (blue). All images show the germlines from the mid pachytene to the diakenesis region, the loop region (in which the cells are in late pachytene or early diplotene) is highlighted by the dotted arc, the mid pachytene region is highlighted by * and late pachytene by **. All germlines are orientated the same way: distal to the left and proximal to the right.

The finding that irradiation rescues CEP-1 expression and pachytene progression in *mpk-1(ga111ts)* mutants indicates that irradiation may activate MPK-1 signaling. To directly test whether this is the case, we took advantage of an antibody that specifically recognises phosphorylated MPK-1 (P-MPK-1) in dissected germlines, and which can be used as a read-out for activated MPK-1 [11], [27], [28]. In wild type worms MPK-1 shows a distinctive phosphorylation pattern: phosphorylation occurs in early to mid pachytene, is absent in late pachytene and early diplotene, and resumes in oocytes, with highest phosphorylation levels observed in the oocyte closest to the spermatheca [11], [15], [28]. We first confirmed this phosphorylation pattern in unirradiated wild type worms (Figure 9A: the bend region is shown by the arc, mid pachytene by *, and late pachytene by **). We next demonstrated in *lip-1* mutants that P-MPK-1 occurs in late pachytene cells residing in the germline bend as previously reported, indicating that LIP-1-mediated dephosphorylation is responsible for the absence of P-MPK-1 in this region of the germline (for representative images see, Figure 9B and 9C) [15]. We note that *lip-1(lf)* mutants have a reduced level of the MPK-1B germline isoform, which correlates with a lower level of total MPK-1B phosphorylation (Figure S4). Nevertheless, we consistently detected P-MPK-1 in the bend region of *lip-1(gt448*) and *lip-1(zh15*) mutant germlines (Figure 9B and 9C). Given that the bend region only comprises a small part of the germline our cytological data does not contradict our observations of total MPK-1B phosphorylation. To see whether IR induces MPK-1 activation in the germline, we dissected wild type and *lip-1* mutant germlines 2–3 hours following irradiation. Interestingly, unlike unirradiated wild type germlines, irradiated wild type germlines show P-MPK-1 throughout the bend region of the germline (Figure 9D) indicating that MPK-1 is activated in the late pachytene/early diplotene region in wild type germlines following IR. Irradiation of the *lip-1* mutant germlines resulted in no obvious change in P-MPK-1 fluorescence compared to unirradiated *lip-1* mutant germlines (Figure 9E and 9F), indicating that *lip-1* mutation likely results in a high level of MPK-1 phosphorylation which cannot be further enhanced by IR. Taken together, our data indicate that the presence of active MPK-1 in late pachytene germ cells correlates with apoptosis induction and that IR activates MPK-1 signaling in the germline.

**Figure 10:**
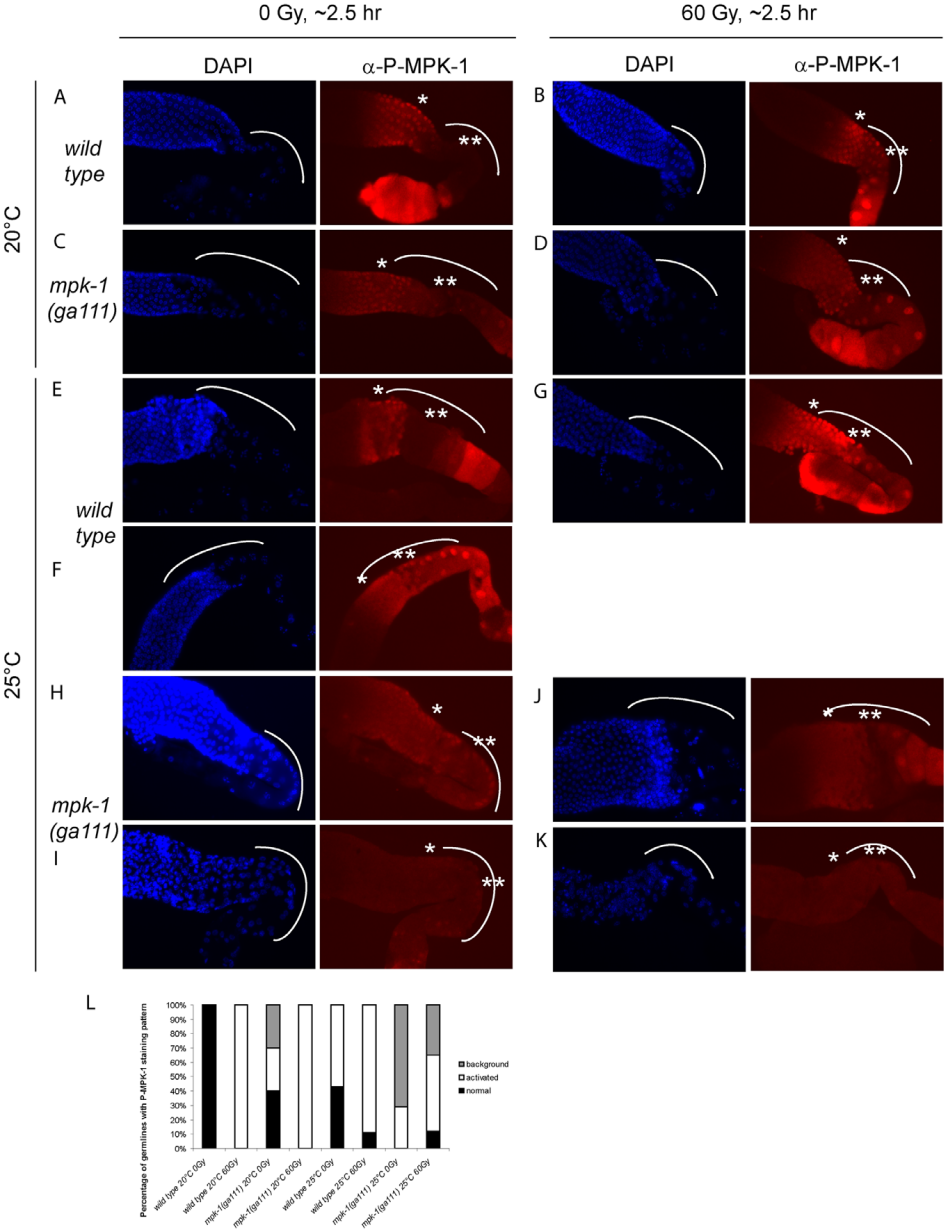
MPK-1 phosphorylation is slightly increased by irradiation in *mpk-1(ga111ts)* mutants raised at 25°C. (A–D) Wild type (A and B) and *mpk-1(ga111ts)* (C and D) mutants raised at 20°C were treated with 0 or 60 Gy, and allowed to recover for 2 hours before germlines were dissected and processed as in Figure 9. (E–K) Wild type (E–G) and *mpk-1(ga111ts*) (H–K) mutants raised at 25°C were treated with 0 and 60 Gy, and allowed to recover for 2 hours before germlines were dissected and processed and presented as in Figure 9. (L) Quantification of the number of germlines with P-MPK-1 staining patterns. ‘normal’ describes a wild type non-irradiated staining pattern, ‘activated’ describes a wild type irradiated staining pattern, and ‘background’ describes very faint or no P-MPK-1 staining. At least four germlines were counted for each genotype and treatment.

Since we observe activation of MPK-1 in wild type germlines following irradiation we were interested in examining whether we could also detect increased phosphorylation of MPK-1 in the *mpk-1(ga111ts)* mutant, which would support our conclusions that MPK-1 is also activated in this mutant. As previously mentioned, the *ga111ts* mutation affects the MEK binding site and is predicted to reduce the rate of MPK-1 activation by MEK [30]. At 20°C this mutant shows no obvious phenotypic defects and this correlates with the almost wild type levels of P-MPK-1 staining observed in germlines from animals raised at 20°C (Figure 10C). The intensity of staining consistently appears to be slightly reduced in the *ga111ts* mutants and a low level persists in the bend region, indicating that MPK-1 may be involved in a negative feedback loop to control its own downregulation. Despite these differences, MPK-1 is clearly activated in *ga111ts* mutants following irradiation as the intensity of staining consistently increases in the bend region and in the developing oocytes (Figure 10D). These findings correlate with the observations that *mpk-1(ga111ts)* mutants raised at 20°C show no phenotypic differences from wild type, including a normal apoptotic response.

We then next examined germlines from animals raised at 25°C. Interestingly, while some wild type germlines showed the characteristic wild type staining pattern without irradiation (Figure 10E), some germlines showed activation of P-MPK-1 in the bend region even without irradiation treatment (Figure 10F, quantified in 10L: ‘normal’ describes the wild type pattern without IR, ‘activated’ describes the wild type pattern following IR, and ‘background’ means no discernable staining). This finding implies that P-MPK-1 may be activated due to stress caused by the elevated temperature. However, upon irradiation more germlines showed phosphorylation in the bend region (Figure 10G, quantified in 10L) indicating that at 25°C MPK-1 still becomes active following irradiation in wild type germlines. Germlines from *mpk-1(ga111ts)* animals raised at 25°C showed two patterns, they either had faint staining throughout the proximal part of the germline (Figure 10H) or they showed a background level (Figure 10I, quantified in 10L). Upon irradiation, more germlines showed some faint staining (Figure 10J) but some still showed background levels (Figure 10K, quantified in 10L), indicating that MPK-1 is activated in these mutants upon IR. However the level of activation in the bend region never approaches wild type levels. These findings correlate with the conclusions we have drawn from our genetic experiments. In the *mpk-1(ga111ts)* mutant worms raised at 25°C MPK-1 activity is greatly reduced resulting in an incomplete pachytene arrest phenotype and very low levels of CEP-1 expression. Upon IR MPK-1 is activated (but not to wild type levels) and this is sufficient to induce pachytene progression and CEP-1 expression but insufficient to induce proper *egl-1* transcription and apoptosis.

### The activation of MPK-1 by IR in DNA damage response mutants

IR induced cell cycle arrest and apoptosis is dependent on signaling by the DNA damage signaling pathway [36]. To test whether MPK-1 activation by irradiation is also dependent on the DNA damage signaling pathway, we assessed P-MPK-1 levels by immunofluorescence in germlines from *atm-1(gk186)* [37], *atl-1(tm853)* [38] and *mrt-2(e2663)* [33] mutants. *atm-1* and *atl-1* encode the homologues of the mammalian phosphatidylinositol 3-kinase proteins ATM and ATR, respectively. These proteins act to sense and signal DNA damage, with ATM responding primarily to double strand breaks and ATR to replication stress. However, there is increasing evidence for cross talk between the two signaling pathways (for recent reviews see [39], [40]). In *C. elegans atm-1* and *atl-1* are required for cell cycle arrest and apoptosis induction in the germline following IR [37], [38]. In addition, *atl-1* is essential for embryogenesis and mutants exhibit mitotic catastrophe and defects in the S-phase checkpoint in mitotic germ cells [38]. *mrt-2* encodes a component of the 9-1-1 complex, which is recruited to sites of DNA damage and is required for full ATR activation [41], [42]. In *C. elegans mrt-2* is required to sense and signal DNA damage in the germline resulting in cell cycle arrest and apoptosis [33], [36].

**Figure 11:**
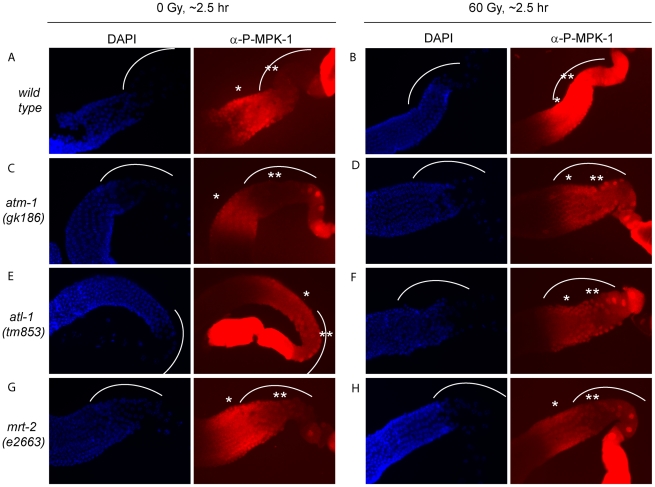
The activation of MPK-1 by irradiation is not dependent on the DNA damage response pathway. (A and B) Wild type, (C and D) *atm-1(gk186)*, (E and F) *atl-1(tm853)*, and (G and H) *mrt-2(e2663)* worms were treated with 0 or 60 Gy, allowed to recover for 2 hours and germlines were then dissected and processed for immunofluorescence and presented as in Figure 9.

P-MPK-1 activation in *atm-1* mutants appeared wild type, with no P-MPK-1 detected in the bend region without IR (Figure 11C) but significant levels following IR (Figure 11D). In contrast, *atl-1* mutants showed clear MPK-1 phosphorylation in the bend region with and without IR treatment (Figure 11E and 11F). P-MPK-1 is also detected in the bend region of *mrt-2* mutants with and without IR treatment. However, the degree of activation is lower than in the middle pachytene region for both treatments (Figure 11G and 11H, compare the ** region with the * region in the images). These findings indicate that *atm-1* and *atl-1* are dispensable for MPK-1 activation by IR. However, the loss of *atl-1* (but not *atm-1*) function in the absence of IR results in the activation of MPK-1 in late pachytene/early diplotene. The activation of MPK-1 could be a result of the high levels of chromosomal instability exhibited by *atl-1* mutants [38] or other defects. Like *atl-1*, *mrt-2* mutants also exhibit chromosomal instability [33] and also have activated levels of MPK-1 in the bend region of the germline. However, the levels of P-MPK-1 are lower than that observed in the *atl-1* mutants and do not significantly increase upon IR, indicating that *mrt-2* may play a role in the activation of P-MPK-1 in response to DNA damage but is not absolutely required.

### CEP-1 and MPK-1 interact in a yeast two-hybrid assay

**Figure 12:**
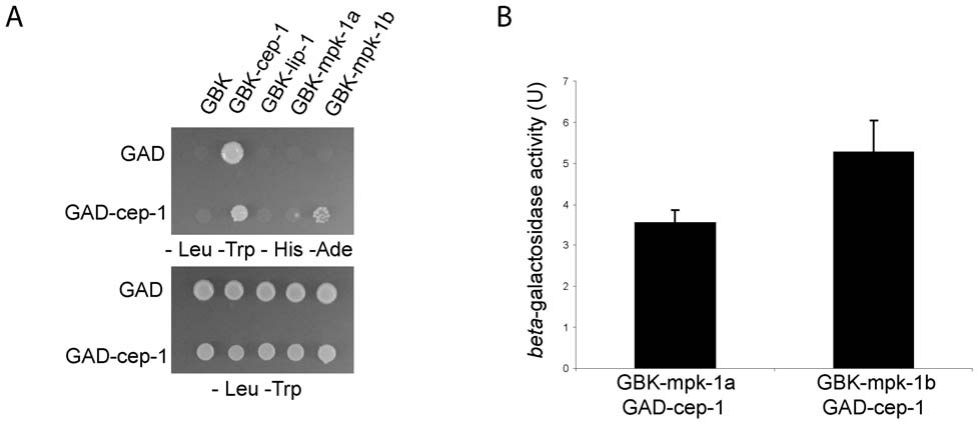
CEP-1 and MPK-1 physically interact. (A) Diploid yeast strains expressing fused Gal4 activation domain (GAD) or Gal4 binding domain (GBK) proteins as indicated were plated onto selective plates (–Leu –Trp –His –Ade) to test for a physical interaction between the fused proteins. –Leu –Trp plates were used to control for growth of the yeast strains. (B) A positive interaction also results in *beta*-galactosidase expression in these yeast strains. The value shown is the measured activity minus the activity of the control strain GAD-*cep-1* GBK-empty. The error bars represent the standard deviation of three replicates.

Given our finding that MPK-1 is active in the late pachytene region of the wild type germline and that a high level of MPK-1 signaling is required for efficient CEP-1 dependent apoptosis induction, we next asked whether MPK-1 could directly activate CEP-1. To test whether CEP-1 could directly interact with MPK-1 we performed a yeast two-hybrid assay. For this, we generated a plasmid containing a fusion between the Gal-4 activation domain (GAD) and *cep-1* cDNA and another set of plasmids with the Gal-4 binding domain (GBK) fused to either *cep-1*, *lip-1*, *mpk-1a*, or *mpk-1b* cDNAs, and generated yeast strains by pairwise matings between GAD and GBK strains. We tested for an interaction by (I) growth on selective media (-His -Ade) and (II) increased *beta*-galactosidase activity (Figure 12A and 12B). As positive controls, we examined known interactions between LIP-1 and the two MPK-1 isoforms, the mammalian ERK, the sevenmaker version of ERK (which binds phosphatases less efficiently), JNK, and p38, as well as between DUSP6 and the MPK-1 isoforms, ERK, sevenmaker, JNK, and p38. In accordance with our *in vivo* dephosphorylation data, both LIP-1 and DUSP6 interact strongly with the MPK-1 isoforms and with ERK, less so with sevenmaker, and not at all with JNK or p38 (Figure S5). As the controls showed the expected interaction we next tested for an interaction between CEP-1 and MPK-1. We observed an interaction between CEP-1 and each of the MPK-1 isoforms, with MPK-1B showing a stronger interaction in both assays (Figure 12A), indicating that CEP-1 and MPK-1A/B do directly interact in yeast cells. While we could not independently confirm this result via co-immunoprecipitation of exogenously expressed CEP-1 and MPK-1 due to an inability to express CEP-1 in mammalian cells, our combined genetic and biochemical evidence suggests that MPK-1 dependent phosphorylation might directly regulate CEP-1 activity.

## Discussion

Using an unbiased genetic screen we found that MAP kinase signaling affects CEP-1 dependent DNA damage induced apoptosis. We provide clear evidence that CEP-1 dependent germ cell apoptosis is increased in mutants with increased MPK-1 activity. Conversely, reduction of MPK-1 activity in *mpk-1(ga111ts)* mutants leads to reduced DNA damage dependent apoptosis. We show that MPK-1 signaling plays important developmental roles in pachytene progression and in regulating CEP-1 expression in pachytene, and a possible direct role in DNA damage induced apoptosis. We postulate that MPK-1 signaling controls DNA damage induced apoptosis through several genetic pathways that all appear to converge on CEP-1. Firstly, MPK-1 signals that germ cells are in late pachytene and that CEP-1 expression can occur. Secondly, MPK-1 signaling regulates GLD-1, which in part could account for the upregulation of CEP-1 expression. Thirdly, MPK-1 is activated in response to IR and this appears to contribute to CEP-1 dependent apoptosis, possibly by direct activation of CEP-1 by MPK-1.

**Figure 13:**
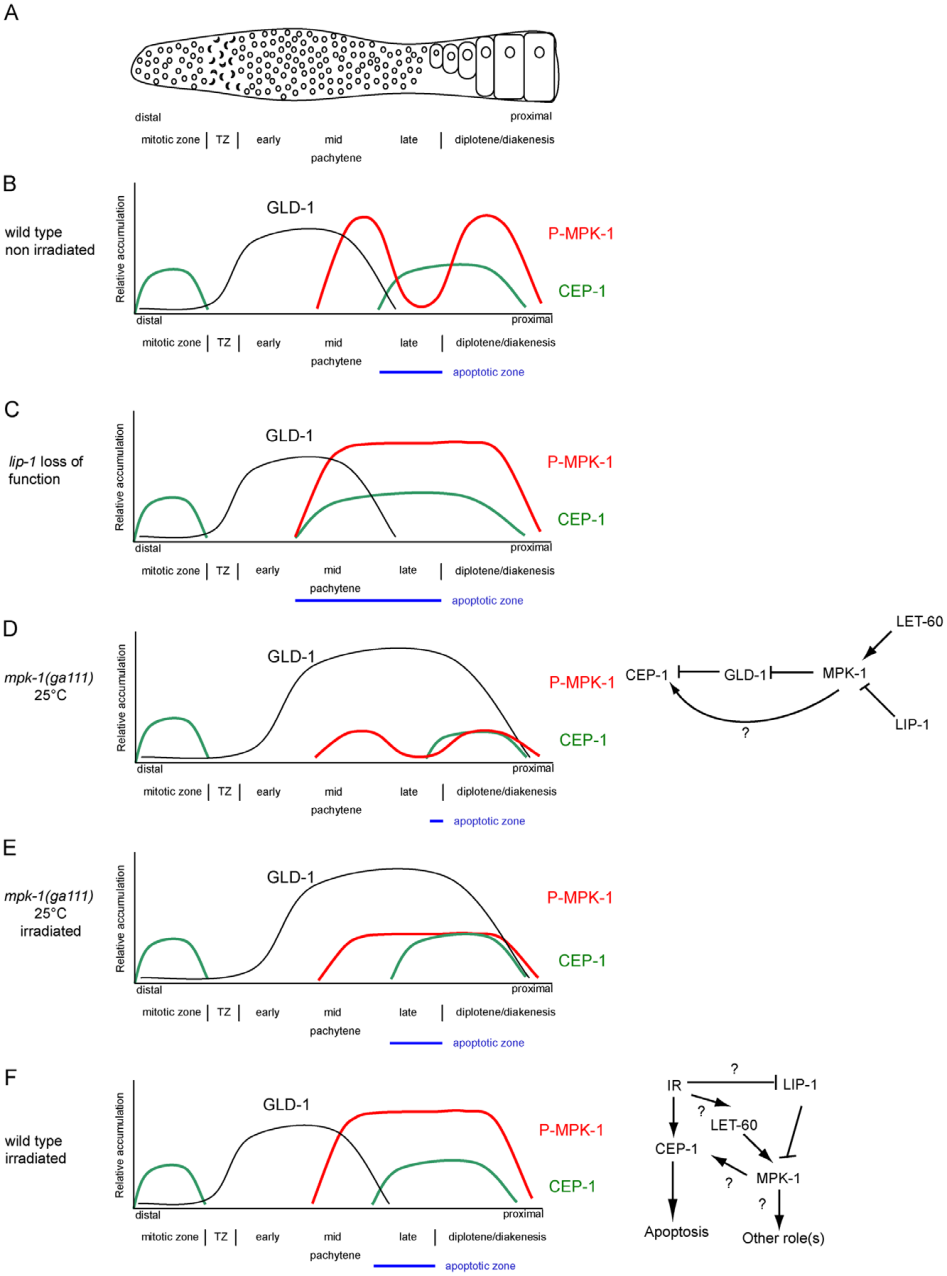
CEP-1 regulation by MPK-1 signaling. (A) Scheme of a hermaphrodite germline. Distal is to the left and proximal oocytes are to the right. (B–F) Schematic drawings depicting the relative accumulation of GLD-1 (black), P-MPK-1 (red), and CEP-1 (green) along the length of the hermaphrodite germline (drawings are aligned with panel A). (B) In wild type unirradiated germlines CEP-1 is expressed in the mitotic zone and in late pachytene and diplotene, GLD-1 is expressed from the transition zone (TZ) to mid pachytene, and MPK-1 is phosphorylated in mid pachytene and in the oocytes. The apoptotic zone (blue) is defined by the expression pattern of CEP-1 in pachytene. (C) In *lip-1* mutant germlines MPK-1 remains phosphorylated throughout late pachytene and early diplotene. CEP-1 is expressed earlier in pachytene resulting in a wider apoptotic zone. (D) In *mpk-1(ga111ts)* worms raised at 25°C phosphorylation of MPK-1 is reduced, CEP-1 expression is also reduced, and GLD-1 levels are high (this study) and persist in the proximal region of the germline [11]. (E). Irradiation of *mpk-1(ga111ts)* worms raised at 25°C results in (partial) activation of P-MPK-1, rescue of CEP-1 expression, but has no effect on GLD-1 protein levels. The regulation of CEP-1 expression by MPK-1 signaling could be due to the effects MPK-1 has on GLD-1 and/or another mechanism (E, right panel). The pattern of GLD-1 expression is adapted from Jones *et al.* 1996 [73] and Schumacher *et al.* 2005 [21]. (F) In wild type irradiated germlines CEP-1 and GLD-1 levels are the same as in unirradiated wild type germlines, but MPK-1 is phosphorylated in the late pachytene. MPK-1 phosphorylation could occur through activation of the upstream signaling pathway or through inhibition of LIP-1. IR-activated MPK-1 may affect CEP-1 directly or it may have another role (F, right panel).

Only cells that are in late pachytene are competent for apoptosis in the *C. elegans* germline. Our results clearly demonstrate that MPK-1 signaling plays a developmental role in establishing apoptotic competency by regulating CEP-1 levels in late pachytene. It does this by regulating the levels of GLD-1, a known translational inhibitor of CEP-1 [21] and by other unknown mechanism(s) independent of GLD-1 levels. Diagrams depicting GLD-1, CEP-1, and P-MPK-1 expression patterns in wild type, *lip-1*, and *mpk-1* mutant germlines are shown in Figure 13A–13E. Our finding that IR can rescue the pachytene arrest phenotype of *mpk-1* worms raised at 25°C has allowed us to examine the role that pachytene progression plays in CEP-1 expression, apoptosis induction, and GLD-1 regulation. Since CEP-1 expression is also rescued in the IR treated *mpk-1* worms it appears that pachytene progression and low MPK-1 activity is sufficient for CEP-1 expression to occur in late pachytene (and to overcome or bypass GLD-1 mediated translational repression). Conversely, enhanced MPK-1 signaling leads to increased CEP-1 expression. Increased CEP-1 expression alone is not sufficient to induce an apoptotic response, as *lip-1(lf)* and *let-60(ga89)* mutants don't show high levels of CEP-1 dependent apoptosis without irradiation (at 20°C). Rather MAP kinase mediated CEP-1 expression primes the cells to respond to a DNA damage signal, and the more cells expressing CEP-1, the greater the apoptotic response. In addition, the rescue of CEP-1 expression in *mpk-1(ga111ts)* mutants raised at 25°C by irradiation does not lead to a wild type apoptotic response or *egl-1* transcriptional induction, indicating that low MPK-1 activity (as shown by the low levels of P-MPK-1 in these germlines (Figure 10)) or restored CEP-1 expression alone are not sufficient to trigger a full apoptotic response. Rather, a normal apoptotic response requires a higher level of MPK-1 activity (see below).

Findings presented in this study indicate that CEP-1 expression in late pachytene, associated with the establishment of apoptotic competency, is under developmental control to ensure that germ cells with damaged DNA or defects in meiotic recombination are culled prior to oogenesis. Interestingly, apoptotic competency in late meiotic prophase seems to be evolutionary conserved: rat and mouse diplotene/diakinetic staged oocytes are more sensitive to IR than oocytes at earlier meiotic stages [43], [44], and p63 expression is also restricted to late pachytene and diplotene staged mouse and human oocytes [45] and pachytene staged mouse spermatocytes [46]. It is thus likely that in mammals p63 expression is subject to analogous developmental control mechanisms to those we have observed for *C. elegans*. While there is no reported evidence that ERK signaling impacts on p63 expression in the mammalian germline, the finding that ERK expression is observed in meiotic prophase in mouse spermatocytes [47], [48] lends weight to the idea that ERK signaling may play a role in regulating apoptosis in the mammalian germline.

Two questions arise from our finding that MPK-1 is phosphorylated in the late pachytene region in response to irradiation: how is MPK-1 phosphorylated in late pachytene, and what role does active MPK-1 play in this region? There are two possible explanations for the first question: either phosphorylated MPK-1 persists in cells progressing from earlier in pachytene (indicating that LIP-1 dependent dephosphorylation is inhibited by IR), or MPK-1 is activated anew by upstream signaling pathway responding to IR. In mammals ERK is activated in response to IR either through the EGF receptor [49]–[51], or by the inhibition of MAPK phosphatases by elevated levels of free radicals (reviewed in [52]–[54]). The finding that P-MPK-1 does not increase beyond a high basal level in *lip-1(lf)* mutants upon IR suggests that the mechanism of IR induced MPK-1 phosphorylation may occur via inhibition of LIP-1. However, it is possible that in *lip-1* mutants maximal MPK-1 activation may already be reached and any enhancement due to upregulation of the signaling pathway is not detectable by immunofluorescence. At this stage our data do not allow us to differentiate between these possibilities. The DNA damage response pathway plays an important role in the cellular response to DNA damaging agents such as IR and it is possible that this pathway is responsible for MPK-1 activation in late pachytene. Our data clearly show that this is not the case as MPK-1 is still phosphorylated in the absence of sensing (*mrt-2*) and signaling (*atm-1* and *atl-1*) gene products (Figure 11). However, the observation that in the absence of *mrt-2* MPK-1 is not strongly phosphorylated in late pachytene upon IR indicates that MRT-2 may be required for full MPK-1 activation.

The second question arising from our observations that MPK-1 is phosphorylated and activated by IR regards its possible role in the damage response pathway. We show that in the absence of strong MPK-1 signaling (in the *mpk-1(ga111)* worms raised at 25°C) apoptosis and *egl-1* transcriptional induction are reduced following IR (Figure 8) despite almost wild type pachytene progression and CEP-1 expression (Figure 6). It is possible that even though the rescue in pachytene progression and CEP-1 expression is almost wild type, they are still not sufficient to induce a wild type apoptotic response in these worms. However, another interpretation of the findings is that the activation of MPK-1 following IR is required for an IR induced cellular response and that it is possible that IR activated MPK-1 facilitates or directly regulates CEP-1 dependent apoptosis. MPK-1 activation occurs within two hours of IR treatment in the late pachytene, where apoptosis occurs [12], [36] and CEP-1 is expressed [21], and this timing correlates with *egl-1* induction (first detected one to two hours post IR [55]). Our finding that MPK-1 and CEP-1 physically interact in a yeast two-hybrid assay lends support to the idea that MPK-1 may directly regulate CEP-1. It will be important to test the possibility that direct MPK-1 dependent phosphorylation is required for CEP-1 activation in future studies. In this study our inability to express CEP-1 in mammalian systems prevented us from using co-immunoprecipitation of heterologous proteins to independently confirm the two-hybrid interactions. Also, our attempts to immunoprecipitate either endogenous CEP-1 or MPK-1 from worm extracts using currently available antibodies failed. Nevertheless, CEP-1 contains a number of putative MAPK phosphorylation and also potential docking sites (data not shown), required to provide high-affinity binding sites between MAPKs and their substrates [56]. The discovery of such consensus sites suggests that CEP-1 could be a possible MPK-1 substrate and future experiments could address this question. Mammalian ERK can phosphorylate and activate p53, leading to cell cycle arrest or senescence [54]. In addition, there is a growing body of evidence indicating that ERK-mediated p53 serine 15 phosphorylation and activation can mediate apoptosis induction following treatment with DNA damaging agents such as doxorubicin [57], [58], cisplatin [59], and UV [60]. We therefore speculate that a conserved mechanism for MPK-1 in mediating damage induced apoptosis through direct CEP-1 phosphorylation may exist in *C. elegans*. If this mechanism does exist it functions in addition or parallel to the activation of CEP-1 by the DNA damage response pathway as induction of the DNA damage response pathway is still required for apoptosis induction in *lip-1* mutants.

Understanding how p53 and p63 are regulated is of vital importance for understanding tumour progression and germline development, respectively. In this work we have begun to dissect the complex relationship between MAPK signaling and p53 dependent apoptosis in the germline. We show that *C. elegans* germline CEP-1 dependent apoptosis is regulated both developmentally and more directly by MAPK signaling in *C. elegans*, and we expect that these mechanisms of regulation could be conserved throughout evolution.

## Materials and Methods

### Strains and *C. elegans* genetics

Worms were maintained at 20°C on NGM plates unless otherwise stated. The strains used were LG I *cep-1(lg12501)* [21], *gld-1(op236)* [21], *atm-1(gk186)* [37], LG III *mpk-1(ga111ts)* [30], *mpk-1(oz140)* [30], *ced-4(n1162)* [61], *mrt-2(e2663)* [33], LG IV *lip-1(zh15)* [16], *lip-1(gt448)* (this study), *let-60(ga89)* [26], *let-60(n1046)* [62], *ced-3(n717)* [63], LG V *egl-1(n1084n3082)* [64], *atl-1(tm853)* [38], CB4856 [65].

Mutants were generated using standard mutagenesis protocols and F2 progeny were screened for enhanced apoptosis 28–30 hr following irradiation using acridine orange staining [21]. *lip-1(gt448)* was backcrossed five times and mapped using standard genetic methods.

For the temperature sensitivity assays, *mpk-1(ga111ts)* and wild type worms were shifted to 25°C as L1 larvae, allowed to develop to the L4 larval or young adult stage, then treated and allowed to recover at 25°C for the time indicated.

### LIP-1 protein expression and dephosphorylation assays

Cos-1 cells were transiently transfected with either *C. elegans* pSG5-LIP-1-Myc or pSG5-LIP-1-KIM-Myc as previously described [66]. The LIP-1 KIM mutant in which both Arg59 and Arg60 were mutated to Ala was generated by overlap extension PCR [67]. Briefly, two independent PCR reactions were performed using pSG5-LIP-1-Myc as template and either primer pair 1 5′-GGCGAATTCTATTTTCAGATGAC-3′ and CCGCCCATTAAAGCGGCTTGAAG-3′, or primer pair 2 5′-CTCTCCTTCAAGCCGCTTTAATG-3′ and 5′-TTCCTCGAGAACTGCAGTTTCG-3′ (nucleotide substitutions are underlined). The PCR products were then mixed and used as template for a third PCR reaction using primers 5′-GGCGAATTCTATTTTCAGATGAC-3′ and 5′-TTCCTCGAGAACTGCAGTTTCG-3′ to generate the mutant reading frame. This amplicon was then subcloned into pSG5-myc as before and verified by DNA sequencing before transfection. As a positive control either pSG5-DUSP1-Myc or pSG5-DUSP5-Myc was used to inactivate ERK, or pSG5-DUSP1-Myc to inactivate p38 and JNK. To activate endogenous ERK cells were serum starved for 16 hrs and then stimulated by addition of 15% FBS. To activate endogenous p38 and JNK cells were exposed to anisomycin (5 µg/ml for 30 min). Following treatment, cells were lysed and proteins analysed by SDS-PAGE and Western blotting using antibodies that detect either the phosphorylated or total amount of the relevant MAPK. Tubulin levels were analysed as a loading control.

### *C. elegans* DNA damage response assays

DNA damage induced apoptosis, radiation (rad) sensitivity, and *egl-1* transcription assays have been previously described [68], [69]. A caesium-137 source (IBL437C, CIS Bio International) was used for the irradiation.

### GLD-1 antibody generation and purification

Rabbit anti-GLD-1 antiserum was raised against recombinant MBP-His-tagged GLD-1 amino acids 155–463 purified using TALON resin (Clontech). For antibody affinity-purification, GST-GLD-1 STAR domain (135–336) fusion protein was coupled to Affi-gel 15 resin (Bio-Rad) according to the manufacturer's guidelines. Rabbit anti-GLD-1 antiserum was incubated with the resin overnight at 4°C, and purified antibody was eluted rapidly using 100 mM glycine pH 2.5 and the pH was neutralised with 1 M Tris, pH 8.8. Once all antibody had apparently been eluted, the resin was then incubated with a further volume of glycine pH 2.5 for 1 hr at 4°C before elution and neutralisation to obtain higher affinity antibodies. Purified antibody was stored in 1% BSA, 10% glycerol and 0.02% thimerosal at −80°C.

### MPK-1 and GLD-1 western blots

Worms were grown until young adults (24 hrs post L4 larval stage), irradiated, and protein was harvested at the indicated times by adding an equal volume of lysis buffer (20 mM Tris HCl pH 8.0, 40 mM Na pyrophosphate, 50 mM NaF, 5 mM MgCl_2_, 100 µM Na vanadate, 10 mM EDTA, 1% Triton X-100, 0.5% deoxycholate). Zirconia/silica beads were added (0.7 mm, BioSpec Products) and the worms were homogenised by beating (3×30 sec, with 30 sec in between) in a Mini-Beadbeater-8 (BioSpec) at 4°C. The homogenate was incubated on ice for 30 min and then centrifuged to remove debris and resulting supernatant was stored at −80°C. An equal amount of protein extract (1 µg for tubulin, 20 µg for total MPK-1, and 40 µg for phosphorylated MPK-1, 10 µg for GLD-1) was boiled in 1× SDS loading buffer and separated on 10% for MPK-1 or 4–12% for GLD-1 Bis-Tris SDS-PAGE gels (Invitrogen). Western blot analysis was performed using ERK (K-23, Santa Cruz 1∶2000, rabbit) and phosphorylated ERK (clone MAPK-YT, Sigma, 1∶2000, mouse) specific antibodies that cross react with MPK-1 and phosphorylated MPK-1 [11], [27], [28] or anti-GLD-1 (1∶500, rabbit, this study) and HRP conjugated secondary antibodies (anti-rabbit-HRP and anti-mouse-HRP, DakoCytomation 1∶2000). Antibody to α-tubulin (DM1A, Sigma, 1∶2000, mouse) was used to control for loading. Band intensity quantification was performed using Image J software.

### Immunofluorescence of dissected germlines

Germlines were extruded into dissection buffer (27.5 mM HEPES pH 7.4, 130 mM NaCl, 53 mM KCl, 2.2 mM CaCl_2_, 2.2 mM Mg Cl_2_, 0.01% Tween20, 0.2 mM levamisole) and fixed with 1.8% (for P-MPK-1 and CEP-1) or 0.5% (for P-H3) formaldehyde (27.5 mM HEPES pH 7.4, 130 mM NaCl, 53 mM KCl, 2.2 mM CaCl_2_, 2.2 mM MgCl_2_) for 5 min (P-MPK-1 and CEP-1) or 4 min (for P-H3). Following freeze cracking, they were post-fixed in 100% methanol (for P-MPK-1 and P-H3) or 50∶50 methanol∶acetone (for CEP-1) for 10 min at −20°C and permeabilised in 0.1% Triton X-100 (for P-MPK-1 and P-H3) or 1% Triton X-100 (for CEP-1) in PBS (4×10 min). Immunofluorescence was performed using antibody to phosphorylated ERK (clone MAPK-YT, Sigma, 1∶100) and anti-mouse AlexFluor-568 (Invitrogen, 1∶500), antibody to CEP-1 ([21], 1∶200) and anti-goat AlexFluor-488 (Invitrogen, 1∶200), or antibody to phosphorylated histone H3 (Ser 10, Millipore, 1∶500) and anti-rabbit AlexFluor-568 (Invitrogen 1∶200). DAPI (1 µg/µl) was used to stain chromatin. Images of P-MPK-1 and CEP-1 stained germlines were taken using an Axioskop 2 (Zeiss) microscope fitted with a RTke camera and accompanying SPOT analysis software (Diagnostic Instruments) using the same exposure settings for each channel. Brightness and contrast of the resulting images were modified to more clearly see the staining patterns but no other changes were made. Images of P-H3 stained germlines were taken using a Leica LMF Spectris microscope and deconvolved using SoftWorx (Applied Precision).

### Yeast two-hybrid assay

Yeast two-hybrid assays were performed as described previously [70]. Briefly, open reading frames encoding *cep-1*, *lip-1*, and human DUSP6 were subcloned into the Gal4 DNA binding domain-fusion (bait) vector pGBK-T7 (Clontech), while the *C. elegans* MAP kinases *mpk-1a/1b*, mammalian ERK2, and the ERK2 *sevenmaker* mutant were subcloned into the Gal4-activation domain-fusion (prey) vector pGAD-T7 (Clontech). pGBK and pGAD fusion constructs were then transformed into yeast strains pJ69-4A and pJ69-4alpha [71] respectively, using the rapid method of Gietz and Woods (2002) [72]. Transformed yeast were selected on auxotrophic media lacking tryptophan (pGBK-fusions) or leucine (pGAD-fusions) respectively. Transformants were mated overnight in 200 µl non-selective YPDA rich medium, of which 50–100 µl of suspended yeast were plated onto dual-selective media lacking leucine and tryptophan. Interactions were probed by growth on media lacking leucine/tryptophan (LT) or leucine/tryptophan/histidine/adenine (LTHA) respectively. Growth on LTHA medium was assessed after 72 hrs of culture at 30°C and considered indicative of an interaction. Semiquantitative analysis of two-hybrid interactions was performed by *beta*-galactosidase assay as described previously [70].

## Supporting Information

Figure S1 Specificity of the anti-CEP-1 antibody. Germlines were dissected from wild type and *cep-1(lg12501)* worms and processed for immunofluorescence using an anti-CEP-1 antibody (green). Nuclei were stained with DAPI (blue).
(TIF)

Figure S2 Specificity of the anti-GLD-1 antibody. Protein was extracted from wild type and *gld-1(q485)* null worms and equal amounts were loaded onto SDS-PAGE gels. GLD-1 was detected by immunoblotting using an anti-GLD-1 antibody and α-tubulin was used to control for loading.
(TIF)

Figure S3 *mpk-1(ga111ts)* mutants show no proliferation defects when raised at 25°C. (A) L4 larval stage worms were irradiated with the specified dose and allowed to recover at 25°C for 24 hours before dissection and staining with DAPI to visualise nuclei. (B) Germlines were dissected from worms 24 hours post L4 larval stage that had been raised at 20°C or 25°C and processed for immunofluorescence using an anti-phospho-histone H3 antibody (red). Nuclei were detected by DAPI (blue). The number of P-H3 nuclei per mitotic zone was counted for each genotype/treatment and the average per germline is shown. At least 16 germlines were counted per genotype/treatment. The error bars represent the standard error of the mean and n.s. stands for ‘not significant’ in an unpaired t-test.
(TIF)

Figure S4 *lip-1(lf)* mutants have reduced levels of MPK-1B protein that is hyperphosphorylated. Protein was extracted from young adult worms (24 hours post L4 larval stage) and equal amounts were loaded onto SDS-PAGE gels. Activated MPK-1 was detected by an anti-phosphorylated-ERK antibody, total MPK-1 by an anti-ERK antibody, and α-tubulin was used to control for loading.
(TIF)

Figure S5 LIP-1 and DUSP6 physically interact with MPK-1A, MPK-1B, and ERK but not JNK and p38. (A) Diploid yeast strains expressing fused Gal4 binding domain (GBK) and Gal4 activation domain (GAD) proteins as stated were plated onto selective media (–Leu –Trp –His –Ade) to test for interaction between the fused proteins. –Leu –Trp plates were used to control for growth of the strains. (B) *Beta*-galactosidase activity of the same yeast strains. The activity shown is the measured activity minus the activity of the control strains GBK-*lip-1* GAD-empty or GBK-DUSP6 GAD-empty. The error bars represent the standard deviation for three replicates.
(TIF)
