# Supplementary figures and images for: Regulation of Caenorhabditis elegans p53/CEP-1–Dependent Germ Cell Apoptosis by Ras/MAPK Signaling
Source: PLoS Genet. 2011 Aug 25;7(8):e1002238. doi: 10.1371/journal.pgen.1002238 (PMC3161941; doi:10.1371/journal.pgen.1002238)

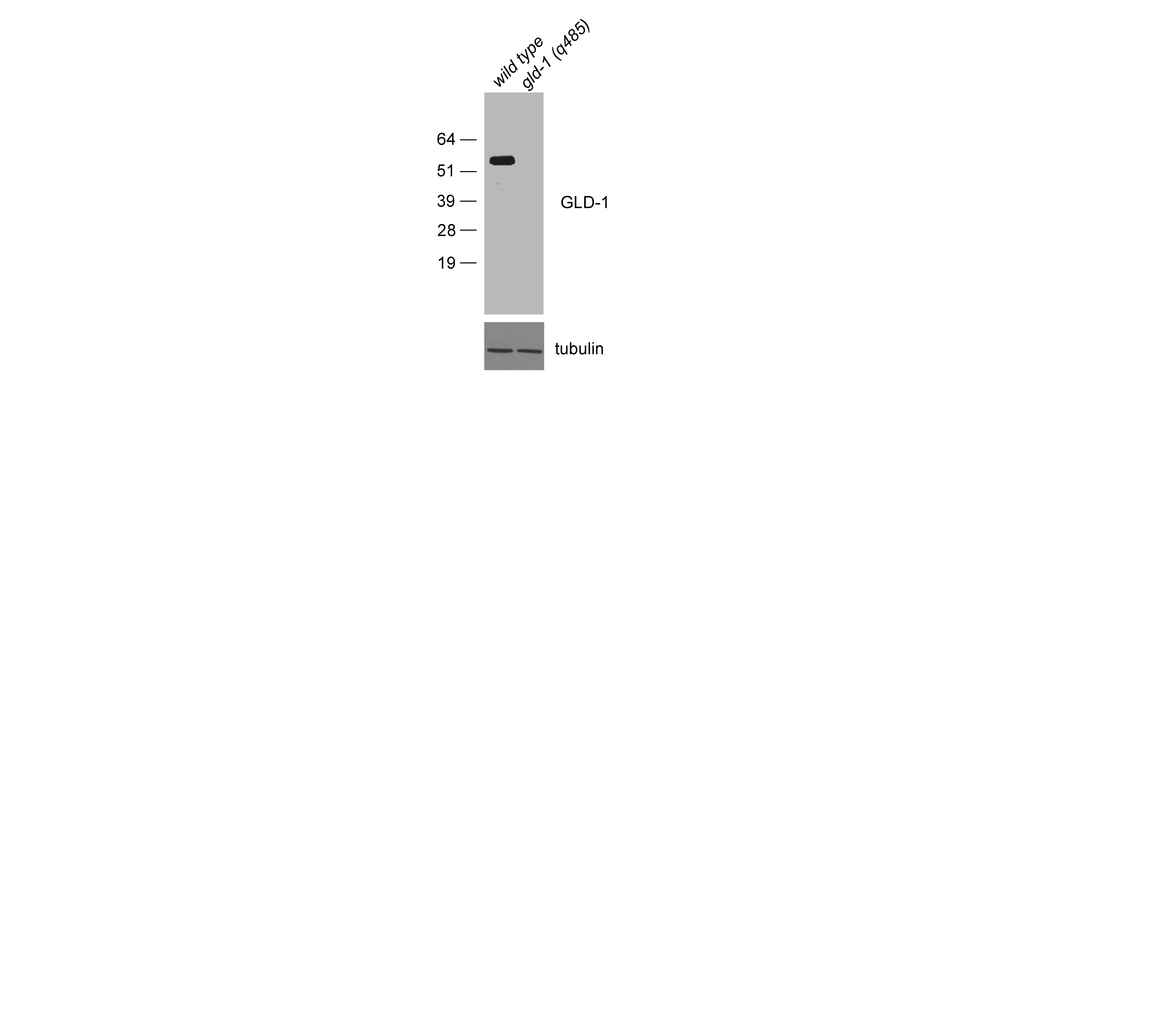

Supplement: Figure S2 — Specificity of the anti-GLD-1 antibody. Protein was extracted from wild type and gld-1(q485) null worms and equal amounts were loaded onto SDS-PAGE gels. GLD-1 was detected by immunoblotting using an anti-GLD-1 antibody and α-tubulin was used to control for loading. (TIF) [file pgen.1002238.s002.tif]

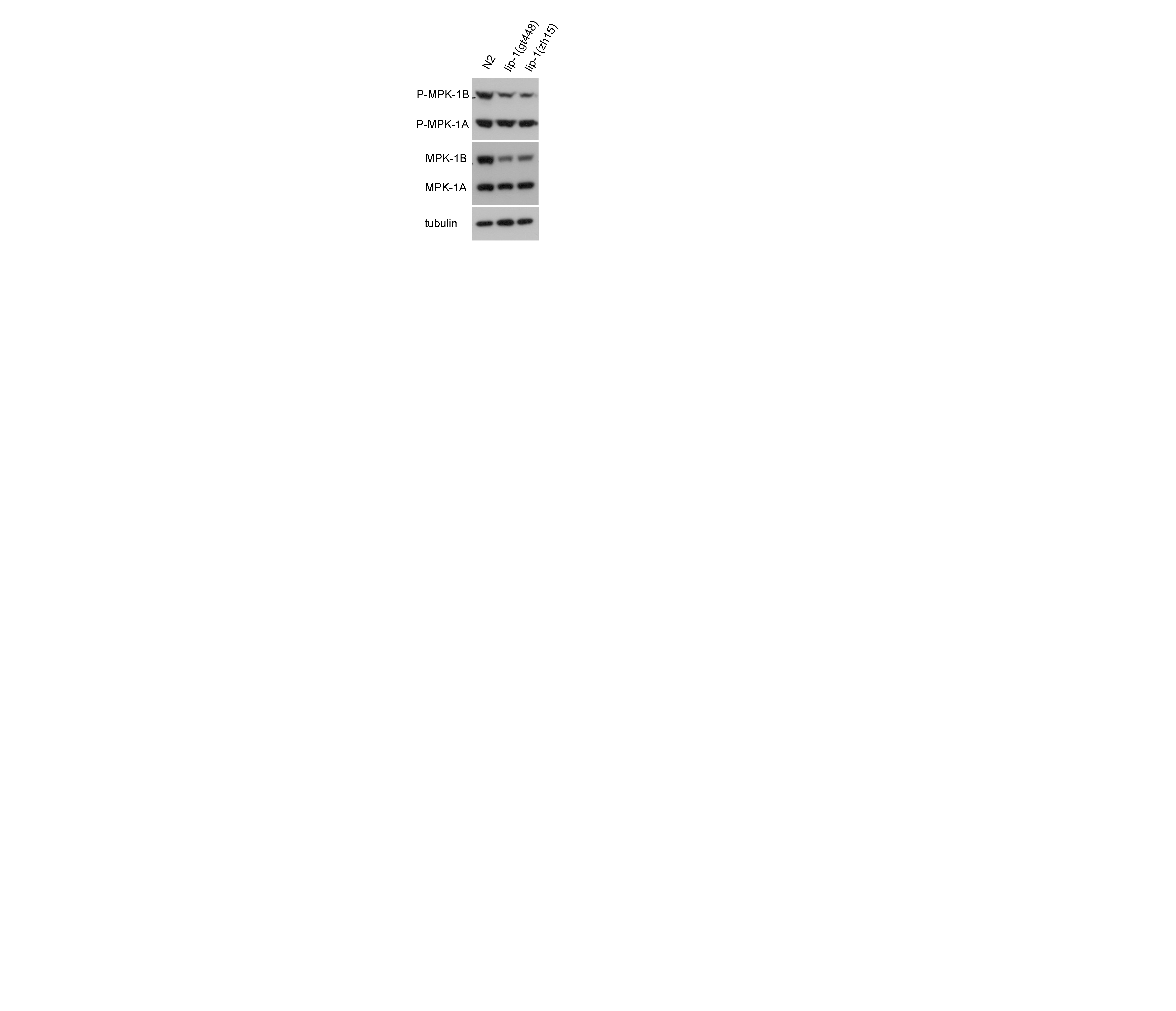

Supplement: Figure S4 — lip-1(lf) mutants have reduced levels of MPK-1B protein that is hyperphosphorylated. Protein was extracted from young adult worms (24 hours post L4 larval stage) and equal amounts were loaded onto SDS-PAGE gels. Activated MPK-1 was detected by an anti-phosphorylated-ERK antibody, total MPK-1 by an anti-ERK antibody, and α-tubulin was used to control for loading. (TIF) [file pgen.1002238.s004.tif]
